# Taxonomy and Phylogeny of Novel and Extant Taxa in Pleosporales Associated with *Mangifera indica* from Yunnan, China (Series I)

**DOI:** 10.3390/jof8020152

**Published:** 2022-02-01

**Authors:** Er-Fu Yang, Saowaluck Tibpromma, Samantha C. Karunarathna, Rungtiwa Phookamsak, Jian-Chu Xu, Zhen-Xiong Zhao, Chathurika Karunanayake, Itthayakorn Promputtha

**Affiliations:** 1 Department of Biology, Faculty of Science, Chiang Mai University, Chiang Mai 50200, Thailand; erfuyang@163.com; 2 Center for Yunnan Plateau Biological Resources Protection and Utilization, College of Biological Resource and Food Engineering, Qujing Normal University, Qujing 655011, China; samanthakarunarathna@gmail.com; 3 Master of Science Program in Applied Microbiology (International Program), Faculty of Science, Chiang Mai University, Chiang Mai 50200, Thailand; 4 Cifor-Icraf China Program, World Agroforestry Centre, Kunming 650201, China; jomjam.rp2@gmail.com (R.P.); jxu@mail.kib.ac.cn (J.-C.X.); 5 Centre for Mountain Futures (CMF), Kunming Institute of Botany, Kunming 650201, China; 6 Department of Environmental Science, Faculty of Resource and Environment, Yunnan Agricultural University, Kunming 650201, China; zzx2677133117@Gmail.com; 7 Department of Botany, The Open University of Sri Lanka, Nawala 10250, Sri Lanka; kokar@ou.ac.lk; 8 Research Center in Bioresources for Agriculture, Industry and Medicine, Chiang Mai University, Chiang Mai 50200, Thailand; 9 Environmental Science Research Center, Faculty of Science, Chiang Mai University, Chiang Mai 50200, Thailand

**Keywords:** 1 new genus, 4 new species, 6 new records, Baoshan, Honghe, mango, plant-associated microfungi, saprobic fungi

## Abstract

Pleosporales is the largest fungal order with a worldwide distribution in terrestrial and aquatic environments. During investigations of saprobic fungi associated with mango (*Mangifera indica*) in Baoshan and Honghe, Yunnan, China, fungal taxa belonging to pleosporales were collected. Morphological examinations and phylogenetic analyses of ITS, LSU, SSU, *rpb2* and *tef1*-*α* loci were used to identify the fungal taxa. A new genus, *Mangifericomes*; four new species, namely *Mangifericomes hongheensis*, *Neomassaria hongheensis*, *Paramonodictys hongheensis*, and *Paramonodictys yunnanensis*; and six new host and country records, namely *Byssosphaeria siamensis*, *Crassiparies quadrisporus*, *Paradictyoarthrinium aquatica*, *Phaeoseptum mali*, *Torula fici*, and *Vaginatispora amygdali*, are introduced. Photoplates, full descriptions, and phylogenetic trees to show the placement of new and known taxa are provided.

## 1. Introduction

Mango (*Mangifera*) belongs to the genus *Mangifera* and the family Anacardiaceae. *Mangifera* contains 69 species, while the most cultivated fruit tree species is *Mangifera indica*, which has over 1000 varieties worldwide, but only a few varieties are cultivated and traded on a large scale [1,2]. *Mangifera indica* is one of the five most economically significant fruit crops worldwide, while it is the second most planted fruit after banana throughout the tropics and subtropics [3,4]. DeCandolle (1884) [5] estimated that mangoes’ cultivation history can trace back 4000 years in India and Southeast Asia, as the fossil evidences discovered show *Mangifera indica* first appeared during the quaternary period. However, the origin of *Mangifera indica* has been debated for years, and some believe mango evolved from several related species in and around Malay Archipelago and China, which may be one of the origins of *Mangifera*, while others insist that mango originated from India and spread worldwide along with the spread of human communities [6,7]. Mango is planted in more than 100 countries, while China, India, and Thailand are ranked the top three countries for cultivated mangoes [8,9].

Mango is subjected to microfungi during pre-harvest and post-harvest activities, while pathogenic fungi can cause serious economic losses to mango worldwide [10,11]. To date, 2236 records of mango-associated microfungi have been documented in the U.S. National Fungus Collections Fungal Database (https://nt.ars-grin.gov/fungaldatabases/, accessed on 25 January 2022), while only 74 records (60 species or 3.3%) belonging to Pleosporales have been found in China, and among these 74 records, *Alternaria*, *Curvularia* (Pleosporaceae), *Phaeosphaerella* (Venturiaceae), *Phoma*, and *Didymella* (Didymellaceae) are ranked as the most frequently found genera from mango [12].

It is very important to screen and study plant-associated microfungi, especially fungi associated with economically important crops, fruits, or other valuable trees. In Asia, especially in China and Thailand, several publications reported fungi in specific hosts. Doilom et al. [13] reported 28 new fungal taxa from *Tectona grandis*, one of the economically important timber trees from northern Thailand. Tibpromma et al. [14] introduced 65 new species of microfungi on Pandanaceae from China and Thailand. Raza et al. [15] reported 32 new fungal species in sugarcane from southern China. Mapook et al. [16] described 130 taxa associated with the invasive weed *Chromolaena odorata* from northern Thailand, and Phukhamsakda et al. [17] reported 50 new species in *Clematis* (Ranunculaceae) from Belgium, China, Italy, Thailand, and the UK. Many saprobic fungi are considered to be host-specific, where these fungi are found only on specific hosts, such as the mangrove saprobes [18]. Moreover, the “host-specificity” is probably the most significant single factor used in estimating global fungal numbers [19]. Roy [20] suggested the terms “host shift,” which only describes the fungi shifting to closely related host taxa, i.e., host tracking coevolution, and “host jumping” which is used for fungi jumping to unrelated or geographically associated hosts. Senwanna et al. [21] reported that *Muyocopron dipterocarpi* probably jumped from *Dipterocarpus tuberculatus* to an introduced host (rubber). In this study, we assume that mango-associated saprobes also probably jumped from various hosts in subtropical to tropical regions, mainly by the agricultural activities.

The order Pleosporales was first proposed by Luttrell [22] as the largest and most diverse order in the class Dothideomycetes (Ascomycota), with 91 families and more than 400 genera [23,24,25]. The sexual morphs are characterized by perithecioid ascomata, ostiolar, some with papillate apex, with or without periphyses, cellular pseudoparaphyses, fissitunicate, bitunicate asci, with ocular chambers or apical ring, wrapped various shapes of ascospores, with pigmentation and septation, with or without sheath, whereas the asexual morphs are usually coelomycetous but sometimes can be hyphomycetous [26,27,28,29,30,31]. Pleosporales mainly occur as saprobic fungi on dead leaves or stems in terrestrial or aquatic environments [26,32,33]. Pleosporales also can be endophytes, epiphytes, parasites of green leaves or stems, and lichenicolous [26,34,35,36].

In this study, we introduce one new genus, four new species and six new host or new country records in Pleosporales collected from mango based on morphological characteristics and multilocus phylogenetic analyses. Full descriptions, color photoplates of macro- and micromorphological characteristics, and phylogenetic trees to show the placement of new and known taxa are provided.

## 2. Materials and Methods

### 2.1. Sampling and Isolation

Specimens were collected from mango plantations and forest areas in Baoshan and Honghe of Yunnan, China. Each sample was placed in a separate Ziplock bag or envelopes together with collection details (collection site, GPS information, collecting date) and transported to the mycology laboratory at Kunming Institute of Botany, Chinese Academy of Sciences. Micro-morphological characteristics were observed and captured by a digital camera (Canon EOS 600D, Canon Inc., Tokyo, Japan) mounted on a compound microscope (Nikon ECLIPSE Ni, Nikon., Tokyo, Japan). Measurements of microstructures were obtained by the Tarosoft (R) Image Frame Work program, while further processing was done in Adobe Photoshop CS3 Extended v. 10.0 (Adobe^®^, San Jose, CA, USA).

Single spore isolation was carried out following the methods outlined in Senanayake et al. [37] by using potato dextrose agar (PDA) and incubating at 27 °C for 12–48 h. Germinated conidia or ascospores were observed with a stereo microscope, transferred to new PDA plates, and incubated at room temperature for 1 week. Culture characteristics were observed after 2 weeks. Specimens were deposited in the herbarium of the Kunming Institute of Botany Academia Sinica (HKAS), while living cultures are maintained at the Kunming Institute of Botany Culture Collection (KUMCC). Index Fungorum numbers were registered as described in Index Fungorum [38].

### 2.2. DNA Extraction, PCR Amplification and Sequencing

Genomic DNA were extracted from fungal mycelium by using Biospin Fungus Genomic DNA Extraction Kit-BSC14S1 (BioFlux^®^, Beijing, China), following the manufacturer’s instructions. The extracted DNA was stored at 4 °C for the Polymerase Chain Reaction (PCR), while a part of the DNA was maintained at −20 °C for long-term storage. The PCR mixture contains 8.5 µL of double-distilled water (ddH_2_O), 12.5 µL of 2×Power Taq PCR MasterMix (mixture of EasyTaqTM DNA Polymerase, dNTPs, and optimized buffer, Beijing Bio Teke Corporation (Bio Teke), China), 1 µL of each forward and reverse primers (10 pmol), and 2 µL of DNA. The internal transcribed spacer (ITS) region was amplified with the primers ITS4 and ITS5, the 18s small subunit (SSU) region amplified by primers NS1 and NS4 [39], the nuclear ribosomal 28s large subunit (LSU) region amplified by the primers LROR and LR5 [40], the partial RNA polymerase II subunit (*rpb2*) region with primers fRPB2-5F and fRPB2-7cR [41], and the partial translation elongation factor 1-alpha (*tef1-α*) gene with primers EF1-983F and 2218R [42]. The conditions for PCR of ITS, SSU, LSU, and *tef1-α* genes constituted an initial denaturation step of 2 min at 95 °C, followed by 35 cycles of 30 s at 95 °C, 50 s at 55 °C, 1 min at 72 °C, and a final denaturation step of 10 min at 72 °C. For the *rpb2* (fRPB2-5F and fRPB2-7cR) gene, the initial denaturation occurred at 95 °C for 3 min; denaturation at 95 °C for 45 s, annealing at 57 °C for 50 s, and extending 90 s at 72 °C for 35 cycles; and extending at 72 °C for 10 min. PCR products were sent to Beijing Bio Teke Corporation for purification and sequencing. To ensure the accuracy of the sequencing, the above methods were repeated to obtain extra sequences for new fungal colonies.

### 2.3. Phylogenetic Analyses

Sequence data both reverse and forward generated in this study were assembled using the Geneious (Restricted) 9.1.2 (https://www.geneious.com, accessed on 25 January 2022) and subjected to BLASTn searches in the nucleotide database of GenBank (http://blast.ncbi.nlm.nih.gov/, accessed on 25 January 2022) to determine their most probable closely related taxa. Single-gene sequence alignments were made with the server version of MAFFT (www.ebi.ac.uk/Tools/mafft, accessed on 25 January 2022) [43] and edited manually in BioEdit 7.2.3 [44]. The uninformative gaps and ambiguous regions were removed by trimAL v1.2 (http://trimal.cgenomics.org, accessed on 25 January 2022) and combined multi-genes sequencing manually in BioEdit. The fasta files were transferred to PHYLIP (for ML) and NEXUS (for BI) format in Alignment Transformation Environment (ALTER) online program [45]. The maximum likelihood analysis (ML) was generated on the CIPRES Science Gateway v.3.3 (http://www.phylo.org/portal 2, accessed on 25 January 2022 [46]) selecting RAxML-HPC2 on XSEDE (8.2.12) [47], with GTRGAMMA substitution model with 1000 bootstrap iterations. The Bayesian analysis performed by using MrBayes v.3.2.2 [48]. The best models of evolution were estimated by using MrModeltest v. 2.3 [49] and PAUP v. 4.0b10 [50]. Bayesian analyses of six simultaneous Markov chains were run for 1,000,000 to 50,000,000 generations (depending on individual fungal groups) and trees were sampled at one tree every 100th or 1000th generation. Phylogenetic trees were visualized using FigTree v1.4.0 [51], and the trees were edited by Microsoft PowerPoint and inserted reliable bootstrap support values from ML and BI.

## 3. Results

### Taxonomy and Phylogenetic Results

Massarinaceae Munk, Friesia 5 (3–5): 305 (1956).

***Vaginatispora*** K.D. Hyde, Nova Hedwigia 61: 234 (1995).

Index Fungorum number: IF 27644; Faces of Fungi number: FoF 00828.

**Type species**: *Vaginatispora aquatica* K.D. Hyde, Nova Hedwigia 61: 235 (1995).

**Figure 1:**
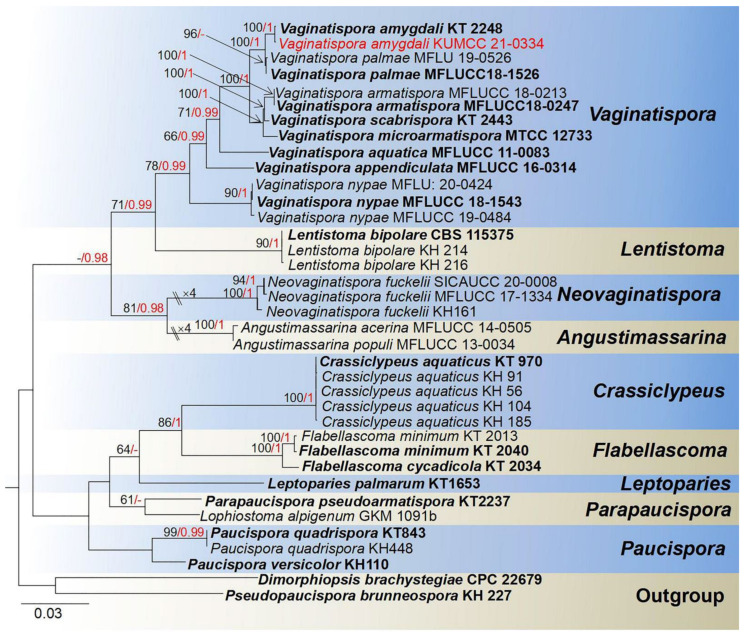
Phylogram generated from Maximum likelihood analysis based on a combined SSU, LSU, *rpb2*, and ITS sequence datasets. Related sequences were taken from Devadatha et al. and Hyde et al. [57,60]. The 37 strains are included in the combined gene analyses’ 3621 total characters, including gaps (SSU: 1–1057 bp, LSU: 1058–1924 bp, *rpb2*: 1925–2959 bp, ITS: 2960–3621 bp). Tree topology of the ML analysis was similar to the BI. The matrix had distinct alignment patterns, with the final ML optimization likelihood value of −14,834.894356 (ln). All free model parameters were estimated by RAxML model, with 279 distinct alignment patterns and 40.11% undetermined characters or gaps. Estimated base frequencies were as follows: A = 0.256878, C = 0.226899, G = 0.272251, T = 0.243972, with substitution rates AC = 1.942312, AG = 4.354437, AT = 1.541678, CG = 1.334482, CT = 9.764709, AND GT = 1.000000. The gamma distribution shape parameter alpha = 0.712530, and the Tree-Length = 1.210702. The final average standard deviation of split frequencies at the end of total MCMC generations calculated as 0.009954 in BI analysis. The species determined in this study are indicated in red. Bootstrap values greater than 60% (ML, left) and Bayesian posterior probabilities (BI, right) greater than 0.95 are given at the nodes; hyphens (-) represent support values less than 60% in ML/0.95 in BI.

**Notes**: *Vaginatispora* was introduced by Hyde [52], with *V. aquatica* as the type species, which is known as saprobic on submerged wood in Australia and initially placed in Massarinaceae. Later, Zhang et al. [53] revealed morphological characteristics and phylogenetic affinity with Lophiostomataceae. Thambugala et al.’s [54] investigations of morphological characteristics and phylogeny proved *Vaginatispora* as a separate genus within Lophiostomataceae. Lately, *Massarina armatispora* was recognized as *Vaginatispora armatispora* [55], and *V. fuckelii* had transferred to the new genus *Neovaginatispora* [56]. The sexual morphs are characterized by elongate ostiolar neck, massarina-like ascospores, with fusiform, hyaline, 1-septate, and wrapped by entire sheath, while asexual morphs have not been reported [52,57,58], and the phylogeny of *Vaginatispora* is shown in Figure 1. Recently, there are eight records in Index Fungorum [38]. *Vaginatispora* species have been collected from submerged wood or terrestrial plant habitats, covering the range of Australia, India, Japan, and Thailand [52,55,56,57,59,60].

**Figure 2:**
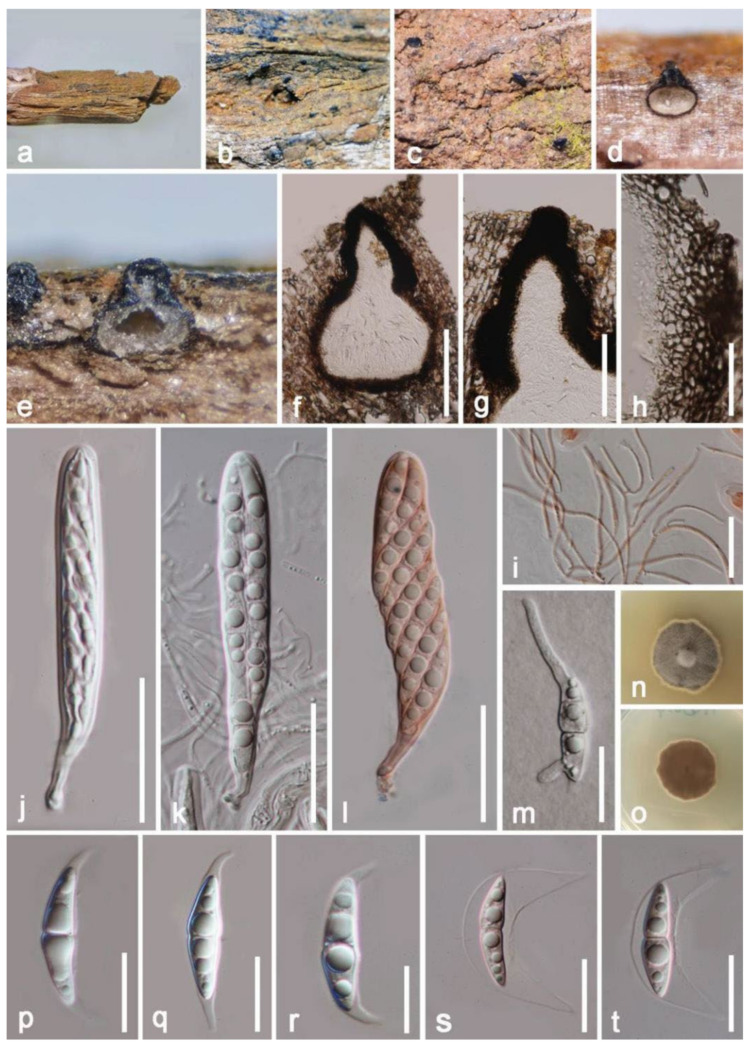
***Vaginatispora amygdali*** (HKAS 122195). (**a**) Appearance on natural substrate; (**b**,**c**) erumpent neck scattered on host surface; (**d**–**f**) vertical section of ascomata; (**g**) prolonged neck; (**h**) peridium; (**i**) pseudoparaphyses stained by congo red; (**j**,**k**) asci; (**l**) asci stained by congo red; (**m**) germinated ascospore; (**n**,**o**) colony on PDA from above and reverse; (**p**–**r**) ascospores; (**s**,**t**) ascospores stained by congo red. Scale bars: (**f**) = 200 μm; (**g**) = 100 μm; (**h**,**j**–**l**) = 30 μm; (**i**,**m**,**s**,**t**) = 20 μm; (**p**–**r**) = 15 μm.

***Vaginatispora amygdali*** A. Hashim., K. Hiray., and Kaz. Tanaka, Studies in Mycology 90: 179 (2018) (Figure 2).

Index Fungorum number: IF 823145.

*Saprobic* on decayed branch of *Mangifera indica*. **Sexual morph**: *Ascomata* (excluding neck) 230–370 × 250–300 μm ($\bar{x}$ = 250 × 280 μm, *n* = 20), pyriform, immersed with erumpent neck, solitary, brown to black, uniloculate, visible black fusiform apical parts scattered on surface, with ostiole at the center. *Ostiole* canal 180–210 μm ($\bar{x}$ = 200 μm, *n* = 15) high, 80–120 μm ($\bar{x}$ = 100 μm, *n* = 15) diameter, prolonged, black, laterally compressed, straight to slight flexuous, and thick-walled. *Peridium* 33–53 μm wide, with several layers, around equal thickness at the base and side, comprising by *textura angularis* cells, hyaline to brown outwardly, outer layers fusing with host. *Hamathecium* 1–2 μm wide, filiform, septate, branched, filamentous, anastomosed, pseudoparaphyses. *Asci* 76–92 × 10–16 μm ($\bar{x}$ = 84 × 13 μm, *n* = 20), 8-spored, oblong, bitunicate, cylindrical to subcylindrical, short with club shape pedicel, hyaline, straight to bent, with a distinct ocular chamber at the immaturity, round at apical. *Ascospores* 26–30 × 4–6 μm ($\bar{x}$ = 27 × 6 μm, *n* = 20), overlapping, biseriate, fusiform, 1-septate at the center, constricted at the septum, conical at both ends, with oil droplets, and with mucilaginous sheath and appendages at both ends. **Asexual morph**: Undetermined.

**Culture characteristics**: Ascospore germinated within 18 h, moderately growing on PDA, reaching 1–20 mm diameter after two weeks at 27 °C, grayish brown to white at the margin, circular, effuse to low convex, indistinctly striate, entire edge to above; white at the margin, reddish brown to pale brown to reverse, without pigments produced from PDA.

**Material examined**: China, Yunnan Province, Baoshan city, Longling county, on decayed branch of *Mangifera indica*, (99°16′80″ E, 25°12′23″ N, 800 m), 27 December 2019, E.F. Yang, MB014 (Herb. HKAS 122195), living culture, KUMCC 21-0334. Genebank numbers; ITS: OL348393, SSU: OL348391, LSU: OL348392, *rpb2*: OL690510.

**Notes**: *Vaginatispora amygdali* was first introduced by Hashimoto et al. [56], collected and isolated from the endocarp of *Amygdalus persica* in Japan. Our isolate *V. amygdali* KUMCC 21-0334 was morphologically similar to member of *Vaginatispora* and *Leptoparies*. However, based on phylogenetic analyses of combined SSU, LSU, *rpb2* and ITS sequence data, our isolate clusters together with *V. amygdali* (KT 2248, holotype) with high bootstrap support (Figure 1). The BLASTn results of LSU, ITS, and SSU of our strain gives 99% similarity with *V. amygdal* (KT 2248). Therefore, based on morphology and phylogeny, we introduce and describe our strain as *V. amygdali* from China on a dead branch of *Mangifera indica*. This is a new host and a new country record (Table 1).

Melanommataceae G. Winter (as “Melanommeae”), Rabenh. Krypt.-Fl., Edn 2 (Leipzig) 1.2: 220 (1885)

***Byssosphaeria*** Cooke, Grevillea 7: 84 (1879).

Index Fungorum number: IF 711; Faces of Fungi number: FoF 00765.

**Type species**: *Byssosphaeria keitii* (Berk. and Broome) Cooke (1879).

**Figure 3:**
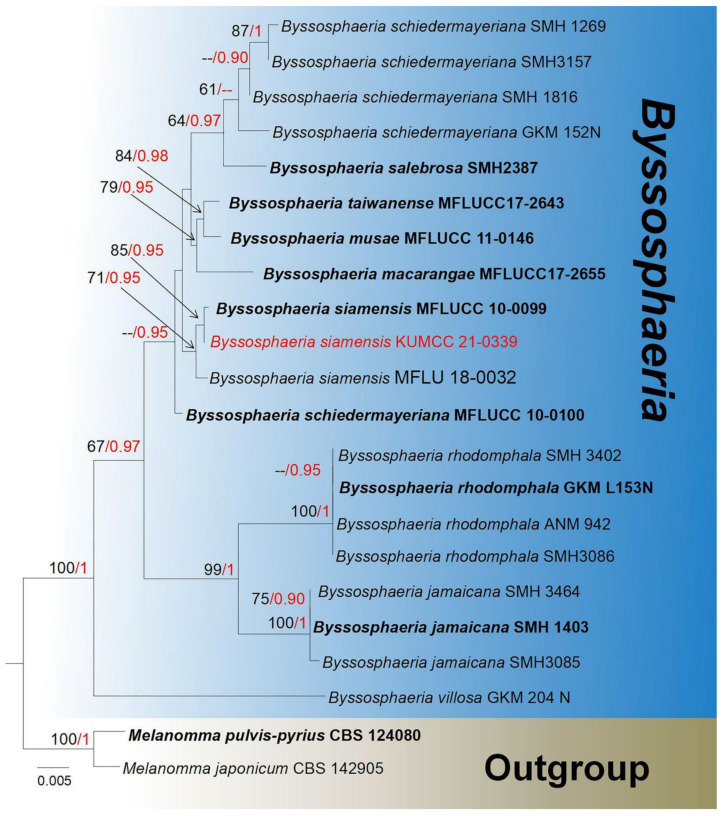
Phylogram generated from maximum likelihood analysis based on a combined LSU, SSU, *tef1-α,* and ITS sequence datasets. Related sequences were taken from Tennnakoon et al. [74]. The 22 strains are included in the combined gene analyses’ 3062 total characters, including gaps (LSU: 1–1019 bp, SSU: 1020–1902 bp, *tef1-α*: 1903–2848 bp, ITS: 2583–3062). Tree topology of the ML analysis was similar to the BI. The matrix had distinct alignment patterns, with the final ML optimization likelihood value of −6168.360196 (ln). All free model parameters were estimated by RAxML model, with 268 distinct alignment patterns and 42.29% undetermined characters or gaps. Estimated base frequencies were as follows: A = 0.246680, C = 0.238985, G = 0.279987, T = 0.234349, with substitution rates AC = 4.527914, AG = 12.745822, AT = 3.626608, CG = 5.279649, CT = 66.381523, GT = 1.000000. The gamma distribution shape parameter alpha = 0.020000, and the Tree-Length = 0.192774. The final average standard deviation of split frequencies at the end of total MCMC generations was calculated as 0.009321 in BI analysis. The species determined in this study are indicated in red. Bootstrap values greater than 60% (ML, left) and Bayesian posterior probabilities (BI, right) greater than 0.90 are given at the nodes; hyphens (-) represent support values less than 60% in ML/0.95 in BI.

**Notes**: *Byssosphaeria* is a widespread genus and was first introduced by Cooke and Plowright [71], with *B. keithii* as the type species, and later placed in Melanommataceae [72]. In morphology, *Byssosphaeria* was easily distinguishable from the similar genus *Herpotrichia*, with trabeculate pseudoparaphyses and presence of a subiculum [73]. The generic type of *Byssosphaeria* was characterized by superficial ascomata, with yellow to orange or red flat apices around the ostiole, with brown to black appendage, and ascospores wrapped by hyaline sheath [61,74]. Species of *Byssosphaeria* are distributed in tropical areas, especially in Asia, and known as a wide range of hosts, such as saprobic on fallen wood, bark, and leaves, and only sexual morphs have been introduced [61,72,74,75]. Recently, there are 38 records in Index Fungorum [38]. The phylogeny of *Byssosphaeria* is shown in Figure 3.

**Figure 4:**
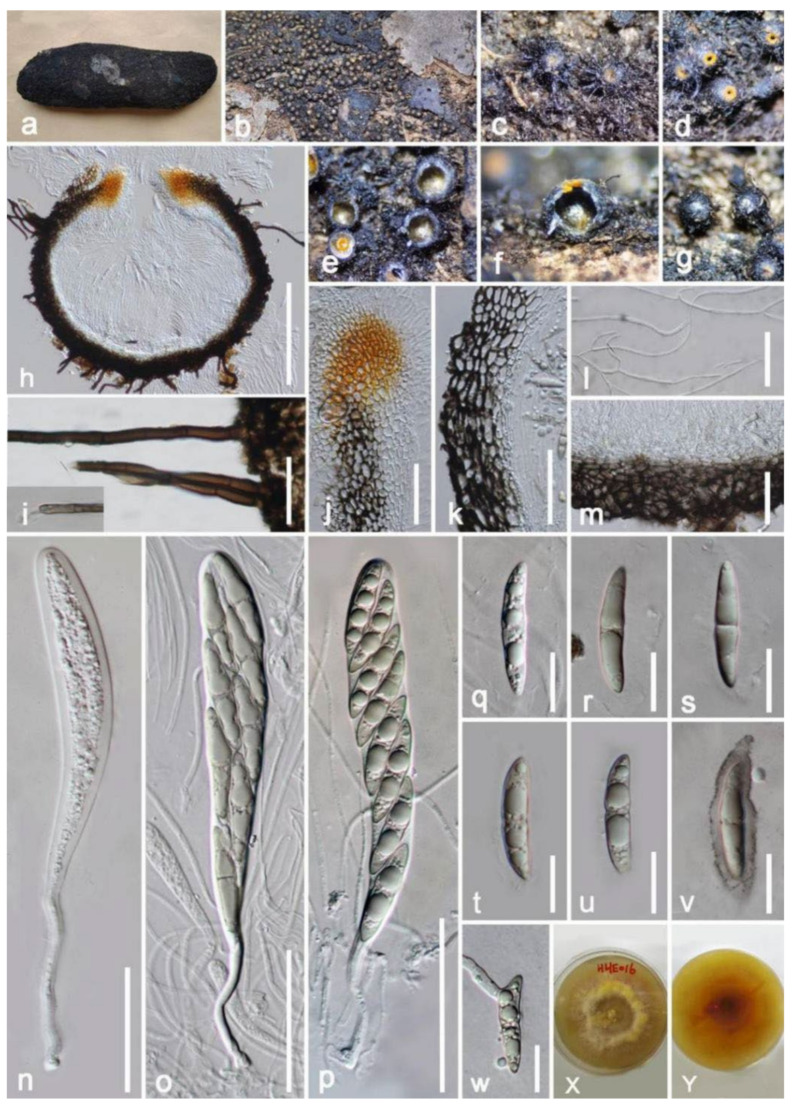
***Byssosphaeria siamensis*** (HKAS 122197). (**a**) Ascomata on decayed endocarp of *Mangifera indica*; (**b**–**d**) close-up of ascomata; (**e**) horizontal section of ascomata; (**f**) vertical section of ascomata; (**g**) bottom of ascoma; (**h**) section of ascomata; (**i**) setae; (**j**) peridium near ostiole; (**k**) peridium at side; (**l**) pseudoparaphyses; (**m**) peridium at base; (**n**–**p**) immature and mature asci; (**q**–**v**) ascospores; (**w**) germinated ascospore; (**x**,**y**) colony on PDA with pigment. Scale bars: (**h**) = 200 μm; (**j**,**k**,**m**,**o**,**p**) = 50 μm; (**i**,**l**,**n**) = 30 μm; (**q**–**w**) = 15 μm.

***Byssosphaeria siamensis*** Boonmee, Q. Tian and K.D. Hyde, Fungal Diversity 74: 283 (2015) (Figure 4).

Index Fungorum number: IF 551430; Faces of Fungi number: FoF 01026.

*Saprobic* on decayed endocarp of *Mangifera indica*. **Sexual morph**: *Ascomata* 290–430 μm ($\bar{x}$ = 390 μm, *n* = 10) high, 440–480 μm ($\bar{x}$ = 420 μm, *n* = 10) diameter, globose to subglobose, superficial, with flat base, uniloculate, dark brown, scattered to gregarious, conspicuous at the surface, black, hairy, ostiole at center, with pore-like opening, surrounded by yellowish to orange disc. *Setae* 4–6-μm wide, filiform, dark-brown to black, apex round, septate. Ostiole surrounded by yellowish to orange, opening when mature. *Peridium* 30–50-μm wide, thick-walled, of equal thickness, multilayered, outer layers dark brown to black, somewhat flattened cells of *textura angularis*; inner layers 7–11-μm wide, hyaline cells of *textura prismatica*. *Hamathecium* 1.5–3-μm wide, composed of dense, branched, septate, pseudoparaphyses, anastomosed, filamentous. *Asci* 140–190 × 16–25 μm ($\bar{x}$ = 184 × 22 μm, *n* = 20), 8-spored, bitunicate, long pedicellate (40–50-μm long) with club-like, apical, rounded, with a small ocular chamber. *Ascospores* 28–33 × 5–7 μm ($\bar{x}$ = 30 × 6 μm, *n* = 20), overlapping, 1–2 seriate, uni- to biseriate, fusiform, subhyaline to greenish brown, slightly constricted at the central septum, conical at each end, 1-septate, guttules, surrounded by a hyaline sheath. **Asexual Morph**: Undetermined.

**Culture characteristics**: Ascospores germinated on PDA within 20 h, rapid-growing on PDA medium, and reached 15 diameter after one week at 27 °C, circular, effuse, fimbriate, fluffy to feathery, medium dense to dense, white to yellowish, ultimately appearing uneven orange on upper surface with maturity, reverse yellowish at the margin, gradually changing to reddish at the center, producing yellow pigments in PDA.

**Material examined**: China, Yunnan Province, Honghe Menglong village, on decayed endocarp of *Mangifera indica*, (102°50′11″ E, 23°41′01″ N, 500 m), 22 December 2020, E.F. Yang, HHE016 (Herb. HKAS 122197), living culture, KUMCC 21-0339. Genbank numbers; ITS: OL413024, LSU: OL413016, SSU: OL413010, *tef1-α*: OL739466.

**Notes**: Our isolate fits well with the concept of *Byssosphaeria*, with globose to subglobose, superficial ascomata, setose, pore-like ostiole, surround by yellowish to orange disc, 8-spored in asci, long pedicel, ascospore oblong fusiform, 1–3-septate, wrapped in a sheath [71,72]. The ITS BLASTn results of our strain show 100% similarity with *Byssosphaeria siamensis* KUMCC 21-0339. In addition, the BLASTn results of SSU, LSU, and *tef1-α* indicated above 99% similarity with *B. siamensis* (MFLUCC 10-0099, MFLU 18-0032), which was established by Boonmee et al. [61]. The multi-gene (SSU, LSU, *tef1-α* and ITS) phylogenetic analysis placed our strain together with *B. siamensis* (MFLUCC 10-0099, MFLU 18-0032) (Figure 3). Therefore, our isolate was identified as *B. siamensis* with new host and country records based on morphology and phylogeny (Table 1).

***Mangifericomes*** E.F. Yang and Tibpromma, gen. nov.

Index Fungorum number: IF 559283; Faces of Fungi number: FoF 10595.

**Etymology**: Name reflects the host plant *Mangifera indica* from which the holotype was collected.

**Type species**: *Mangifericomes hongheensis* E.F. Yang and Tibpromma.

*Saprobic* on dead or decaying branch, leaves or wood of terrestrial, **Sexual morph**: *Ascomata* immersed or semi-immersed, globose to subglobose, sometime visible apical black neck effuses to host surface, dark brown to black, with or without ostiolate. *Peridium* comprised by thick, dark-brown walled cells of *textura angularis* to *globosa*. *Hermathecium* comprises filiform, hyaline, thick-walled, septate, branched, with some oil droplets, pseudoparaphyses. *Asci* 8-spored, bitunicate, cylindrical-clavate, pedicellate, apically rounded with an ocular chamber. *Ascospores* muriform, ellipsoid, often have one widened apical part in each, 7–11-transversely transversally septate, and 5–8 longitudinal septa, slightly or strongly constricted at the septum, wrapped by a gelatinous sheath. **Asexual morph**: Undetermined.

**Figure 5:**
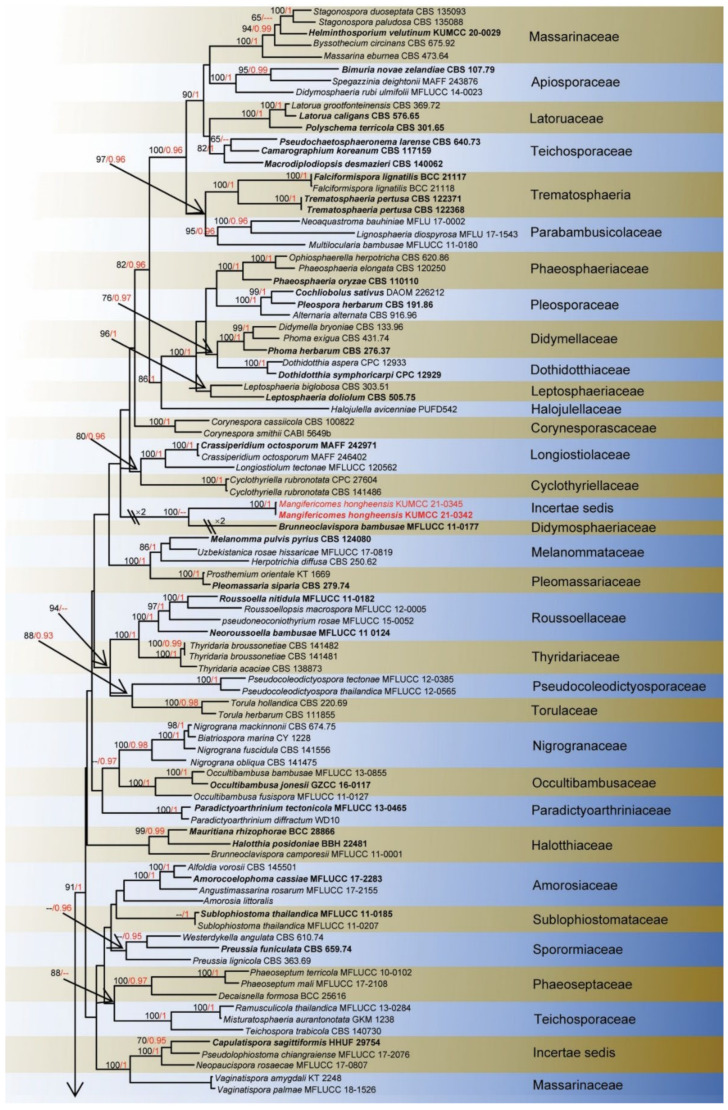

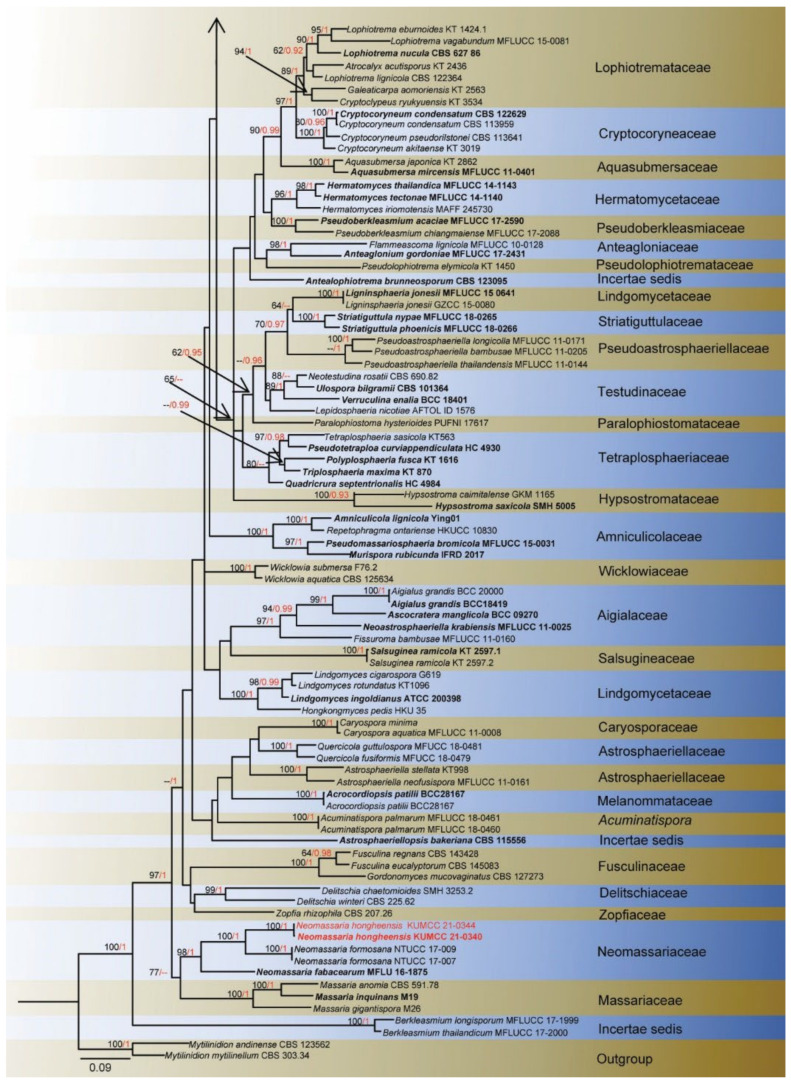
Phylogram generated from maximum likelihood analysis based on a combined LSU, SSU, *rpb2*, *tef1-α,* and ITS sequence datasets. Related sequences were taken from Zhang et al. [24], Ariyawansa et al. [30], and Hongsanan et al. [81]. The 180 strains are included in the combined gene analyses’ 4531 total characters, including gaps (LSU: 1–880 bp, SSU: 881–1913 bp, *tef1-α*: 1914–2840 bp, *rpb2*: 2841–3878 bp, ITS: 3879–4531), Tree topology of the ML analysis was similar to the BI. The matrix had distinct alignment patterns, with the final ML optimization likelihood value of −117,367.689956 (ln). All free model parameters were estimated by RAxML model, with 2953 distinct alignment patterns and 32.22% undetermined characters or gaps. Estimated base frequencies were as follows: A = 0.247310, C = 0.247387, G = 0.270304, T = 0.234998, with substitution rates AC = 1.384565, AG = 3.688216, AT = 1.401064, CG = 1.072637, CT = 7.214852, GT = 1.000000. The gamma distribution shape parameter alpha = 0.324973, and the Tree-Length = 19.839324. The final average standard deviation of split frequencies at the end of total MCMC generations was calculated as 0.009444 in BI analysis. The species determined in this study are indicated in red. Bootstrap values greater than 60% (ML, left) and Bayesian posterior probabilities (BI, right) greater than 0.90 are given at the nodes; hyphens (-) represent values less than 60% in ML/0.95 in BI.

**Notes**: The new genus *Mangifericomes* was established as a monotypic genus in Pleosporales genera *incertae sedis*, which was isolated from *Mangifera indica* in China. *Mangifericomes hongheensis* as the type species is characterized by ellipsoid and muriform, pale brown to brown ascospores, wrapped in a gelatinous sheath, while the other genera, namely. *Murispora, Phaeoseptum, Halojuella, Julella,* and *Pleospora*, also have muriform, yellow-brown to dark-brown ascospores, with or without a sheath [76,77,78,79]. However, *M. hongheensis* differs in having 7–11-transversely septate and 5–8 longitudinal septa in each spore, oblong, more obtuse, and flatter at both ends, and the pseudoparaphyses are sparse, short-branched, and thick-walled. Furthermore, *M. hongheensis* clustered in an individual group (Figure 5) and separated well with other genera, which has muriform ascospores in phylogenetic trees.

**Figure 6:**
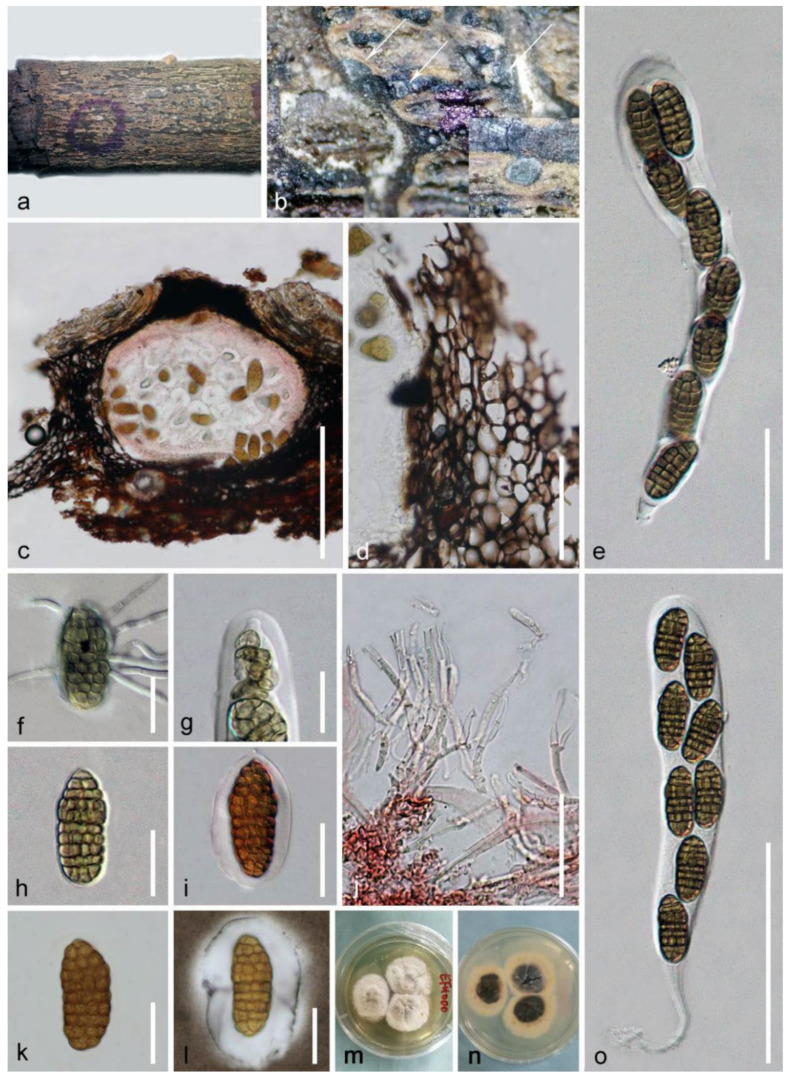
***Mangifericomes hongheensis*** (HKAS 122188, holotype) (**a**) Ascomata on decayed branch of *Mangifera indica*; (**b**) close-up of substrate; (**c**) section of ascoma stained by congo red; (**d**) peridium stained by congo red; (**e**,**o**) asci; (**f**) germinated ascospore; (**g**) ocular chamber; (**h**) brown ascospore; (**i**,**k**) ascospores stained with congo red; (**l**) ascospore stained with Indian ink; (**j**) pseudoparaphyses stained by congo red; (**m**,**n**) colonies on PDA. Scale bars: (**c**) = 150 μm; (**e**,**o**) = 100 μm; (**d**) = 50 μm; (**j**) = 30 μm; (**f**–**i**,**k**,**l**) = 20 μm.

***Mangifericomes hongheensis*** E.F. Yang and Tibpromma, sp. nov. (Figure 6).

Index Fungorum number: IF 559284; Faces of Fungi number: FoF 10596.

**Etymology**: Name reflects the location Honghe where the holotype was collected.

**Holotype**: HKAS 122188.

*Saprobic* on decayed branch of *Mangifera indica*. **Sexual morph**: *Ascomata* 135–250 μm ($\bar{x}$ = 188 μm, *n* = 20) high, 243–326 μm ($\bar{x}$ = 285 μm, *n* = 20) diameter, scattered to gregarious, globose to subglobose, semi-immersed to fully immersed, emerging as dark brown to black raised regions on host surface, with ostiole. *Peridium* 20–35-µm wide, thick-walled, dark brown to black, multilayered, the upper parts in contact with the bark, arranged in a *textura angularis* to *globosa*, and 10–18-µm wide at the middle parts. *Hamathecium* 1.5–2.5-μm wide, hyaline, septate, branched, cellular, pseudoparaphyses, sparse, embedded in a glutinous matrix. *Asci* 140–180 × 20–30 μm ($\bar{x}$ = 165 × 25 μm, *n* = 30), 8-spored, bitunicate, fissitunicate, cylindrical-clavate, with long or minute pedicel, apically rounded, with an ocular chamber. *Ascospores* 30–40 × 12–16 μm ($\bar{x}$ = 35 × 15 μm, *n* = 20), overlapping uni- or biseriate, pale-brown to brown, ellipsoid, slightly bent to straight, muriform, rough-walled, slightly wider near apex, 7–11-transverselly septate, and 5–8 longitudinal septa, slightly constricted at the septum, enclosed in a gelatinous sheath 8–11-µm wide. **Asexual morph**: Undetermined.

**Culture characteristics**: Ascospores germinated within 20 h on water agar, with colonies on PDA reaching 7–12-mm diameter after 1 week on the PDA medium, circular, convex, raised, erose or dentate, moderately dense, above cottony with abundant white, slight gray to white from margin to center; reverse: visible as white loop at the margin, brown to dark in the center, sunken, without pigments produced in PDA.

**Material examined**: China, Yunnan Province, Honghe Menglong village, on decayed branch of *Mangifera indica*, (102°50′11″ E, 23°41′01″ N, 500 m), 24 July 2019, E.F. Yang, EFH000 (Herb. HKAS 1221888, holotype), ex-type, KUMCC 21-0342 = KUMUCC 21-0345. Genbank number; ITS: OL413014, LSU: OL413021, SSU: OL413018, *rpb2*: OL739470, *tef1-α*: OL739469 (KUMCC 21-0342); ITS: OL413017, LSU: OL413019, SSU: OL413023, *rpb2*: OL754591, *tef1-α*: OL754590 (KUMUCC 21-0345).

**Notes**: *Mangifericomes hongheensis* KUMCC 21-0342 was identified as a territorial saprobic fungus associated with a dead branch of *Mangifera indica*. The BLASTn searches of the LSU sequence of *M. hongheensis* KUMCC 21-0342 resulted in 93.67% and 94.31% matches with *Roussoella hysterioides* CBS 125434 and *Paradictyoarthrinium hydei* MFLUCC 17-2512, the ITS showed 83% (455/547, 36 gaps) matches with *Dothideomycetes* sp. PS18-9, and *tef1-α* BLASTn results appeared at 91.5% similarity with *Neoaquastroma bauhiniae* (MFLU 17-055), *Brunneofusispora inclinatiostiola* (GZCC 21-0185), and *Neooccultibambusa kaiyangensis* (CGMCC 3.20404); the *rpb2* BLASTn results indicated 74% similarity with *Dendryphiella salina* (CBS 142.60) and *Pleospora herbarum* (EGS04-188C). The multi-gene analyses show *M. hongheensis* well separated with other genera/families in Pleosporales with high statistical support values (100% ML and 1.00 BI) (Figure 5), and our isolates show completely different ascospores compared with *Brunneoclavispora bambusae* MFLUCC 11-0177 (Didymosphaeriaceae) even though they are relatively closely related in phylogeny [80]. Our new species is very similar to *Halojulella avicenniae* (Halojulellaceae, Pleosporales) in immersed ascomata, muriform ascospores, and gelatinous sheath [77], but *Halojulella avicenniae* differs in having dark brown, thick-walled peridium; consisting of *textura angularis* to *globosa* cells; and having an ascospore often more obtuse and flatter at both ends (6–7 transverselly, 2–3 longitudinal vs. 7–11 transverselly, and 5–8 longitudinal). Therefore, we introduce *Mangifericomes* as a distinct new genus in order Pleosporales, with *Mangifericomes hongheensis* KUMCC 21-0342 as the type species.

Neomassariaceae H.A. Ariyaw., Jaklitsch and Voglmayr, Cryptog. Mycol. 39(3): 367 (2018).

***Neomassaria*** Mapook, Camporesi and K.D. Hyde, in Hyde et al., Fungal Diversity 80: 74 (2016).

Index Fungorum number: IF 827113.

**Type species**: *Neomassaria fabacearum* Mapook, Camporesi and K.D. Hyde, Fungal Diversity 80: 77 (2016).

**Notes**: Initially, *Neomassaria fabacearum* was introduced as the type in a monotypic novel genus *Neomassaria* (Massariaceae) [82], However, Ariyawansa et al. [30] collected a neomassaria-like species (*Neomassaria formosana*) from stem of *Rhododendron* sp. in Taiwan Province of China. The maximum likelihood and Bayesian phylogenetic analyses formed a separate sister group with *Neomassaria fabacearum* and was well-separated with *Massaria* species (Massariaceae); hence, a new family, Neomassariaceae, was established with a single genus, *Neomassaria*, and two species (*Neomassaria fabacearum* and *N. formosana*). Sexual morph of the genus *Neomassaria* is characterized by having immersed, subglobose to globose ascomata, central ostiole, peridium comprising cells of *textura angularis*, pseudoparaphyses, 8-spored, bitunicate oblong to cylindrical, pedicellate asci, containing ellipsoid to fusiform, 1-septate, hyaline ascospores, and with or without a gelatinous sheath [30,82], while asexual morph has not yet been obtained.

**Figure 7:**
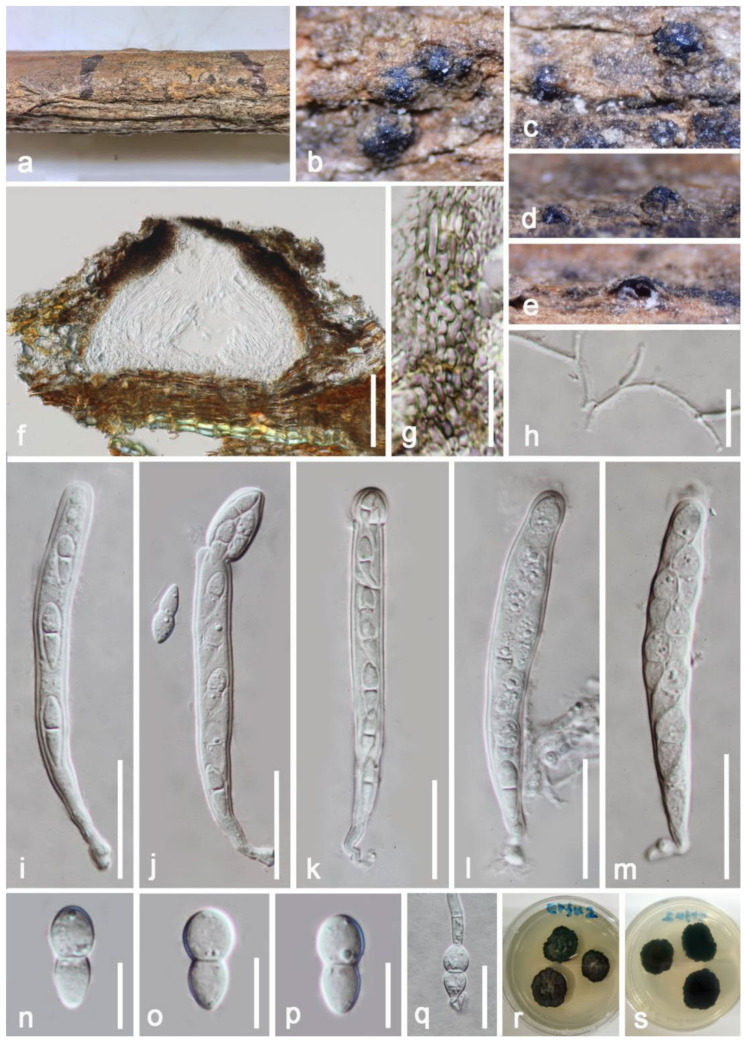
***Neomassaria hongheensis*** (HKAS 122191, holotype). (**a**) Appearance of ascomata on host substrate; (**b**–**d**) close-up of substrate; (**e**,**f**) vertical section of ascoma; (**g**) peridium; (**h**) pseudoparaphyses; (**i**–**m**) asci; (**n**–**p**) ascospores; (**q**) germinated ascospore; (**r**,**s**) colonies on PDA. Scale bars: (**f**) = 100 μm; (**i**–**m**) = 30 μm; (**q**) = 20 μm; (**g**,**n**) = 15 μm; (**n**–**p**) = 10 μm.

***Neomassaria hongheensis*** E.F. Yang and Tibpromma, sp. nov. (Figure 7).

Index Fungorum number: IF 559286; Faces of Fungi number: FoF 10594.

**Etymology**: Name reflects the location Honghe, where the holotype was collected.

**Holotype**: HKAS 122191.

*Saprobic* on decayed branch of *Mangifera indica*. **Sexual morph**: *Ascomata* 160–230 μm ($\bar{x}$ = 190 μm, *n* = 20) high, 220–300 μm ($\bar{x}$ = 260 μm, *n* = 20) diameter, scattered to gregarious, dome-shaped, semi-immersed, appearing as dark black raised regions on host surface, without papillate, with ostiole at center. *Peridium* 30–50-µm wide, composed of several layers, apical areas more darkened in pigments and thicker, exposed, multilayered, the inner layers comprising of hyaline and thick-walled cells of *textura angularis*, and the outer layers almost disappear at base where fused with host. *Hamathecium* 1–2-μm wide, hyaline, anastomosed, filamentous, septate, branched, cellular, embedded in a glutinous matrix, pseudoparaphyses. *Asci* 90–120 × 8–12 μm ($\bar{x}$ = 110 × 10 μm, *n* = 30), 8-spored, bitunicate, 1–2 seriate, fissitunicate, cylindrical-clavate, with a clear pedicel, with a visibly well-developed ocular chamber when young, sometimes markedly constricted near apical region when mature, short pedicellate, sometimes absent. *Ascospores* 14–17 × 4–8 μm ($\bar{x}$ = 15 × 6 μm, *n* = 20), ovoid, uni to biseriate, hyaline, 1-septate in the middle, broadens at upper region, rounded at both ends or acute at the base, granules, smooth-walled, not surrounded by a mucilaginous sheath. **Asexual morph**: Undetermined.

**Culture characteristics**: Ascospore germinated within 20 h on water agar, growing on PDA reaching around 10-mm diameter after 2 weeks incubation at 27 °C above: circular, flat to effuse, rough on surface, wrinkled, dark-brown to black well-defined at margin, dense; reverse: black, without pigments produced in PDA.

**Material examined**: China, Yunnan Province, Honghe Menglong village, on decayed branch of *Mangifera indica*, (102°50′11″ E, 23°41′01″ N, 500 m), 24 July 2019, E.F. Yang, erfu2 (Herb. HKAS 122191, holotype), ex-type, KUMCC 21-0340 = KUMCC 21-0344. Genbank number; ITS: OL477594, LSU: OL423112, SSU: OL423114, *rpb2*: OL754593, *tef1-α*: OL754592 (KUMCC 21-0340); ITS: OL477614, LSU: OL423113, SSU: OL423115, *rpb2*: OL754595, *tef1-α*: OL754594 (KUMCC 21-0344).

Notes: Ariyawansa et al. [30] and Hongsanan et al. [81] were referred for the phylogeny, and LSU, SSU, *tef1-α*, *rpb2* and ITS gene were used for the phylogenetic analyses in Pleosporales. From the phylogenetic trees, we found our isolates (KUMCC 21-0340, KUMCC 21-0344) well separated and closely related with other two species in Neomassariaceae, namely *Neomassaria formosana* (NTUCC 17-007 and NTUCC 17-009) and *N. fabacearum* (MFLU 16-1875), with high statistical support values (ML: 100%, BI: 1, Figure 5). However, these three strains have few differences in morphology. *Neomassaria fabacearum* is characterized by having immersed ascomata, ostiole central, fusiform, ellipsoid to broadly fusiform ascospore surrounded by a gelatinous sheath, comprising two equal cells, narrow in both ends, and initially placed under family Massariaceae [82] *Neomassaria formosana* was established by Ariyawansa et al. [30] and described with immersed ascomata, erumpent, compressed neck, produce fusoid to ellipsoid, oblong or slight flexuous ascospores (20–30 × 3–7 µm), visibly distinct oil droplets in each ascospore but without sheath [30], while our strain has semi-immersed ascomata, widens at the base, without erumpent neck, ascospore (14–17 × 4–8 μm) fusoid, fine at the bottom when it is immature, becomes ovoid later, broadens at upper region, rounded at both ends, distinctly constricted at the septum, without a sheath. In addition, based on BLASTn search, the closest matches of LSU show *Neomassaria formosana* strain NTUCC 17-009 and NTUCC 17-007 (97%, 825/849 bp, 5 gaps), *N. fabacearum* MFLU 16-1875 (95%, 808/850 bp, 5 gaps), and *N. formosana* and *N. fabacearum* only match at 96% in 855 bp. The SSU BLASTn results show our strains mostly overlap with *N. formosana* (NTUCC 17-009 and NTUCC 17-007) and *N. fabacearum* MFLU 16-1875 at around 98% similarity, the BLASTn of *tef1-α* region show our strain KUMCC 21-0340 only has 90% similarity to *N. fabacearum* MFLU 16-1875 and 94% similarity to *N. formosana* strains; in addition, *N. fabacearum* MFLU 16-1875 and *N. formosana* strains just overlapped 89% in *tef1-α* region. We compared the differences of base pairs of *rpb2* from *N. formosana* and our strains, and the similarity was less than 90%, and therefore, based on morphological examinations and phylogenetic analyses, we introduce our strains as a new species *N. hongheensis*.

Parabambusicolaceae Kaz. Tanaka and K. Hiray., Studies in Mycology 82: 115 (2015).

***Paramonodictys*** N.G. Liu, K.D. Hyde and J.K. Liu, Fungal Diversity 100: 90 (2020).

Index Fungorum number: IF 557092; Faces of Fungi number: FoF 06709.

**Type species**: *Paramonodictys solitarius* N.G. Liu, K.D. Hyde and J.K. Liu, Fungal Diversity 100: 91 (2020).

**Figure 8:**
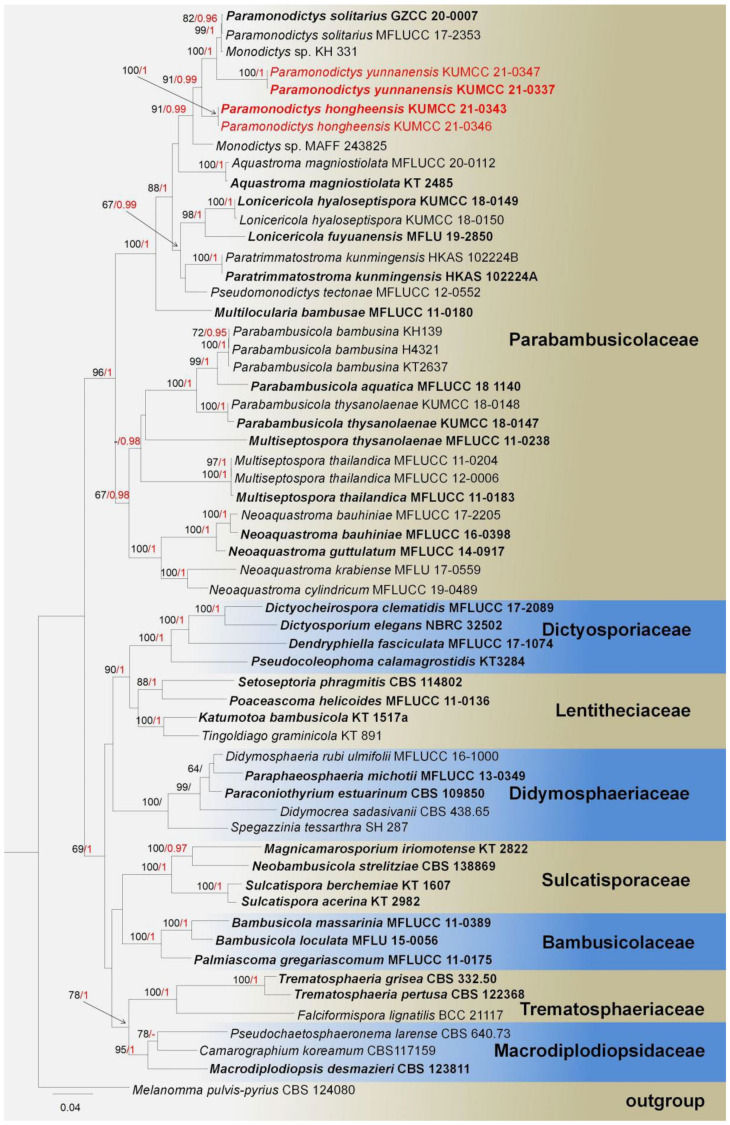
Phylogram generated from maximum likelihood analysis based on a combined SSU, LSU, ITS, and *tef1-α* sequence datasets. Related sequences were taken from Dong et al. and Hyde et al. [83,84]. The 59 strains are included in the combined gene analyses’ 3540 total characters, including gaps (LSU: 1–879 bp, SSU: 880–1907 bp, *tef1-α*: 1908–2841 bp, ITS: 2842–3540 bp). Tree topology of the ML analysis was similar to the BI. The matrix had distinct alignment patterns, with the final ML optimization likelihood value of −27,170.920818 (ln). All free model parameters were estimated by RAxML model, with 1459 distinct alignment patterns and 21.90% undetermined characters or gaps. Estimated base frequencies were as follows: A = 0.235389, C = 0.254603, G = 0.273057, T = 0.236951, with substitution rates AC = 1.064440, AG = 2.450499, AT = 1.326228, CG = 1.102216, CT = 5.326081, GT = 1.000000. The gamma distribution shape parameter alpha = 0.516907, and the Tree-Length = 3.201655. The final average standard deviation of split frequencies at the end of total MCMC generations was calculated as 0.009940 in BI analysis. The species determined in this study are indicated in red. Bootstrap values greater than 60% (ML, left) and Bayesian posterior probabilities (BI, right) greater than 0.95 are given at the nodes; hyphens (-) represent values less than 60% in ML/0.95 in BI.

**Notes**: *Paramonodictys* was first introduced by Hyde et al. [83] with *P. solitarius* as the type species, which is known to be saprobic on decaying wood in terrestrial habitats; later, on another collection, it was reported from freshwater by Dong et al. [84] This genus is morphologically characterized as having superficial black colonies, monoblastic conidiogenous cells, pyriform or clavate, brown to olivaceous brown dictyosporous, subglobose to globose conidia. *Paramonodictys* presently has been investigated only as an asexual morph, and it has a complex placement with *Monodictys* sp. (KH331 and MAFF 243825) in phylogenetic analyses (Figure 8) [84].

**Figure 9:**
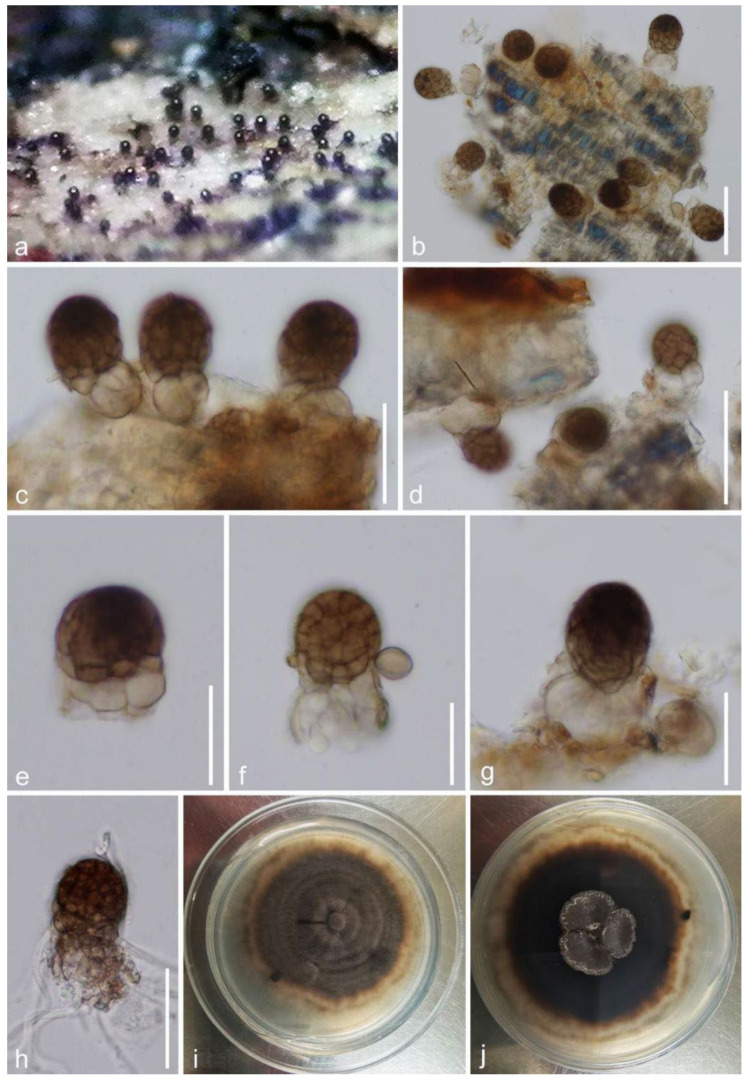
***Paramonodictys hongheensis*** (HKAS 122190, holotype). (**a**) Colonies on surface of bark; (**b**–**d**) colonies on host surface; (**e**–**g**) conidia with conidiogenous cells and conidiophore; (**h**) germinated conidium; (**i**,**j**) colony from above and below. Scale bars: (**b**,**d**) = 50 μm; (**c**,**h**) = 30 μm; (**e**–**g**) = 20 μm.

***Paramonodictys hongheensis*** E.F. Yang and Tibpromma, sp. nov. (Figure 9).

Index Fungorum number: IF 559288; Faces of Fungi number: FoF 10592.

**Etymology**: Name reflects the location Honghe where the holotype was collected.

**Holotype**: HKAS 122190.

*Saprobic* on decayed branch of *Mangifera indica*. **Sexual morph**: Undetermined. **Asexual morph**: Hyphomycetous. *Colonies* on natural substrate scattered or gregarious, effuse, solitary to gregarious, subglobose to obovoid, visible brownish to dark brown spots scattered on the host surface, shiny, easily separating when disturbed. *Conidiogenous cells* 10–15 × 16–25 μm ($\bar{x}$ = 12 × 20 μm, *n* = 20), monoblastic, subhyaline to brown, often subglobose to irregular, smooth or frequently appear vertical septa. *Conidia* 19–26 × 19–22 μm ($\bar{x}$ = 23 × 21 μm, *n* = 30), subglobose to oval, muriform, thick-walled, multicellular with regular or more often irregular septation, apical parts pigments dark brown, lower parts pale-brown to yellow-brown.

**Culture characteristics**: Conidia germinated within 18–20 h on PDA. Colonies rapid-growing on PDA reached about 15-mm diameter, after 1 week at room temperature in natural light, the upper surface of colonies appear circular, umbonate, slightly radial striations, circinate, fluffy, entire edge, initially grayish to pale brown, finally exhibiting dark brown when it matures after 1–2 months. Reverse dark brown, reddish-brown near the margin, sunken at the center, without pigments produced in PDA.

**Material examined**: China, Yunnan Province, Honghe Menglong village, on decayed branch of *Mangifera indica*, (102°50′11″ E, 23°41′01″ N, 500 m), 24 July 2019, E.F. Yang, EFH005 (Herb. HKAS 122190, holotype), ex-type, KUMCC 21-0343 = KUMCC 21-0346. Genbank numbers; ITS: OL436229, LSU: OL436227, SSU: OL436232, *tef1-α*: OL505582 (KUMCC 21-0343); ITS: OL: OL436235, LSU: OL436224, SSU: OL436225, *tef1-α*: OL505583 (KUMCC 21-0346).

**Notes**: In our study, *Paramonodictys hongheensis* KUMCC 21-0343 has a high similarity with *Monodictys melonopa* (PAN 32767) in morphology, which was introduced by Prasher and Verma [85], conidia multicellular, muriform, subglobose to oval, pale-brown in the lower parts, and the conidia of our isolate was smaller than conidia of *M. melonopa* (19–26 × 19–22 μm vs. 26–46 × 19–28 µm); unfortunately, we lack genes of *M. melonopa* to compare it with our isolate. Based on our phylogeny, our new isolates formed a separate branch basal to *Monodictys* sp., *Paramonodicty solitarius*, and *P. yunnanensis* with high bootstrap support in ML and BI (Figure 8). The morphological characteristics of our new isolate and other *Paramonodictys* spp. are very similar [83,84]. In addition, comparison of the nucleotides across the *tef1-α* gene region showed a quite few numbers of base pairs were different compared with *Monodictys* sp. KH331 (difference: 911/948 bp, 3.9%, 0 gaps) and *Paramonodictys solitarius* MFLUCC 17-2353 (890/921 bp, 3.4%, 0 gaps). Therefore, based on conidial morphology comparison and phylogenetic analyses, *Paramonodictys hongheensis* is established as a new species on *Mangifera indica* from China.

**Figure 10:**
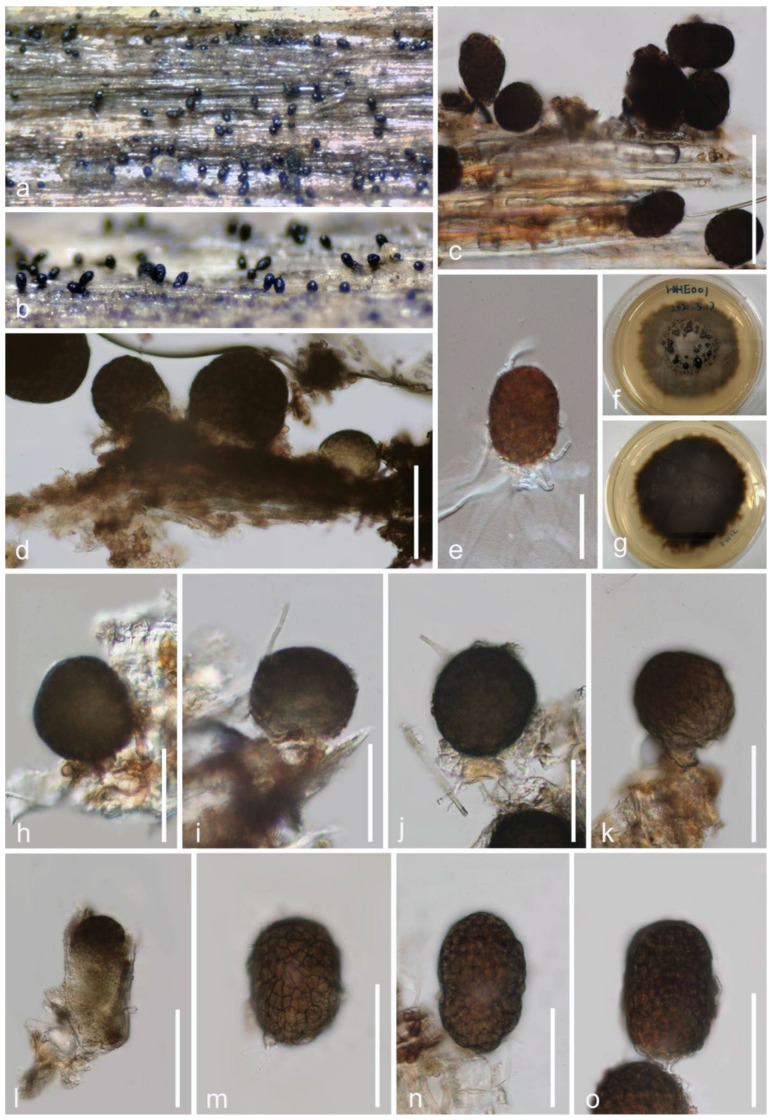
***Paramonodictys yunnanensis*** (HKAS 122189, holotype). (**a**,**b**) Close-up of colonies on substrate; (**c**,**d**) conidia attached on host; (**e**) single germinated conidium; (**f**,**g**) colony from above and below; (**h**–**k**) conidia connect with conidiogenous cells; (**l**–**m**) immature and mature conidia. Scale bars: (**c**) = 100 μm; (**d**,**e**,**h**–**o**) = 50 μm.

***Paramonodictys yunnanensis*** E.F. Yang and Tibpromma, sp. nov. (Figure 10).

Index Fungorum number: IF 559287; Faces of Fungi number: FoF 10591.

**Etymology**: Name reflects the location Honghe where the holotype was collected.

**Holotype**: HKAS 122189.

*Saprobic* on decayed branch of *Mangifera indica*. **Sexual morph**: Undetermined. **Asexual morph**: *Colonies* on substrate effuse, superficial, erect, solitary, obovoid to oblong, visible brownish to dark brown dots scattered on the host surface, easily separating when disturbed. *Mycelium* fully immersed. *Conidiophores* reduced to conidiogenous cells. *Conidiogenous cells* 8–12 μm ($\bar{x}$ = 10 μm, *n* = 20) in high, 9–12 μm ($\bar{x}$ = 11 μm, *n* = 20) in diameter, brownish to reddish-brown, cylindrical. *Conidia* 47–70 × 35–47 μm ($\bar{x}$ = 58 × 41 μm, *n* = 20), obovoid to subglobose, muriform, thick-walled, irregularly multiseptate, brown to olivaceous brown when mature, not in chains.

**Culture characteristics**: Conidia germinated within 18–20 h on PDA. Colonies rapid-growing on PDA reach about 10–15-mm diameter after 2 weeks at room temperature in natural light; colony circular, umbonate, fluffy, gray to brown from above, dark-brown from below, erose to dentate, without pigments produced in PDA.

**Material examined**: China, Yunnan Province, Honghe Menglong village, on decayed branch of *Mangifera indica*, (102°50′11″ E, 23°41′01″ N, 500 m), 22 December 2020, E.F. Yang, HHE001 (Herb. HKAS 122189, holotype), ex-type, KUMCC 21-0337 = KUMCC 21-0347. Genbank numbers; ITS: OL436231, LSU: OL436226, SSU: OL436230, *tef1-α*: OL 505585, *rpb2*: OL505584 (KUMCC 21-0337); ITS: OL436233, LSU: OL436228, SSU: OL436234, *tef1-α*: OL 505586 (KUMCC 21-0347).

**Notes**: The morphological characteristics of *Paramonodictys yunnanensis* sp. nov. fits well with the generic type of *Paramonodictys*. Our new species is similar to *P. solitaries* in having brown to olivaceous-brown and obovoid to oblong conidia; however, the conidiogenous cells of *P. yunnanensis* are shorter than *P. solitarius*, while colonies on PDA have distinct differences with *P. solitarius* MFLUCC 17-2353 (brown, erose margin vs. olivaceous brown, entire edge) [84]. In the NCBI BLASTn of ITS sequence, our strain *P. yunnanensis* (KUMCC 21-0337) highly overlapped with *P. solitarius* (MFLUCC 17-2353 and GZCC 20-0007) at 98.97% and 98.74% similarity, while LSU (874 bp) BLASTn showed high similarity with *Monodictys* sp. (KH 331 and MAFF 243825) at 99.77% and 99.08%, the SSU (1028 bp). The *rpb2* BLASTn result was quite close to *Paramonodictys solitarius* GZCC:20-0007 (99%), while the *tef1-α* showed only 86.5% (837/935 bp, 3 gaps) similarity to *P. solitarius* GZCC 20-0007 and *Monodictys* sp. MAFF 243825. Thus, *P. yunnanensis* is described as a new species in *Monodictys* and *Paramonodictys* complex clade. Moreover, the multi-gene (SSU, LSU, ITS, and *tef1-α*) phylogeny generated herein reveal *P. yunnanensis* at basal clade of *P. solitarius* with well-separated branch with high bootstrap support value in ML and BI (Figure 8). Therefore, our isolate can be described as a new species on *Mangifera indica* from China.

Paradictyoarthriniaceae Doilom, J.K. Liu and K.D. Hyde, Fungal Diversity 72: 133 (2015).

***Paradictyoarthrinium*** Matsush., Matsushima Mycological Memoirs 9: 18 (1996).

Index Fungorum number: IF 554082; Faces of Fungi number: FoF 03933.

**Type species**: *Paradictyoarthrinium diffractum* Matsush., Matsushima Mycological Memoirs 9: 18 (1996).

**Figure 11:**
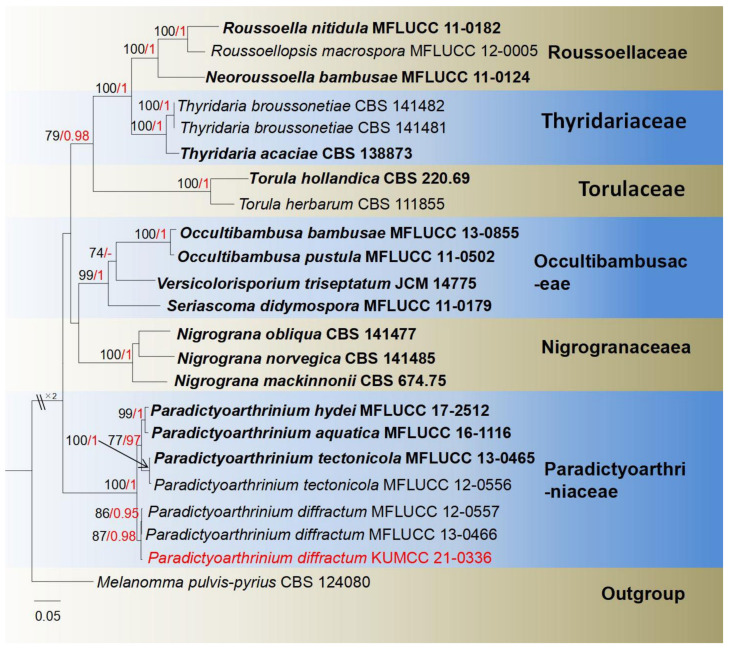
Phylogram generated from maximum likelihood analysis based on a combined LSU, ITS, and *rpb2* sequence datasets. Related sequences were taken from Liu et al. [86]. The 23 strains are included in the combined gene analyses’ 2523 total characters, including gaps (LSU: 1–867 bp, ITS: 868–1447 bp, *rpb2*: 1448–2523 bp). Tree topology of the ML analysis was similar to the BI. The matrix had distinct alignment patterns, with the final ML optimization likelihood value of −14,675.292545 (ln). All free model parameters were estimated by RAxML model, with 1017 distinct alignment patterns and 15.66% undetermined characters or gaps. Estimated base frequencies were as follows: A = 0.251864, C = 0.243671, G = 0.279161, T = 0.225303, with substitution rates AC = 1.434366, AG = 3.862103, AT = 1.470306, CG = 0.996484, CT = 9.230078, GT = 1.000000. The gamma distribution shape parameter alpha = 0.697593, and the Tree-Length = 1.990250. The final average standard deviation of split frequencies at the end of total MCMC generations was calculated as 0.008418 in BI analysis. The species determined in this study are indicated in red. Bootstrap values greater than 60% (ML, left) and Bayesian posterior probabilities (BI, right) greater than 0.95 are given at the nodes; hyphens (-) represent values less than 60% in ML/0.95 in BI.

**Notes**: The monotypic genus *Paradictyoarthrinium* was established by Matsushima [63] with *P. diffractum* as the type species, which was collected from dead twigs in a stream in South Africa. This genus contains only *P. aquatica*, *P. diffractum*, *P. hydei* and *P. tectonicola*, in Index Fungorum [38], while those hydrophilous or terricolous taxa were collected from China and Thailand [86,87]. The genus is characterized by superficial, powdery colonies, conidiophores macronematous, unevenly dictyoseptate, muriform, conidiogenous cells blastic, integrated, terminal, determinate, subglobose to irregular, brown to black conidia congregate on the top, sometimes developing in branched chains, with 1–2 short chains [64,86]. Sexual morphology has not been documented in this genus. The phylogeny of *Paradictyoarthrinium* and closely related genera is shown in Figure 11.

**Figure 12:**
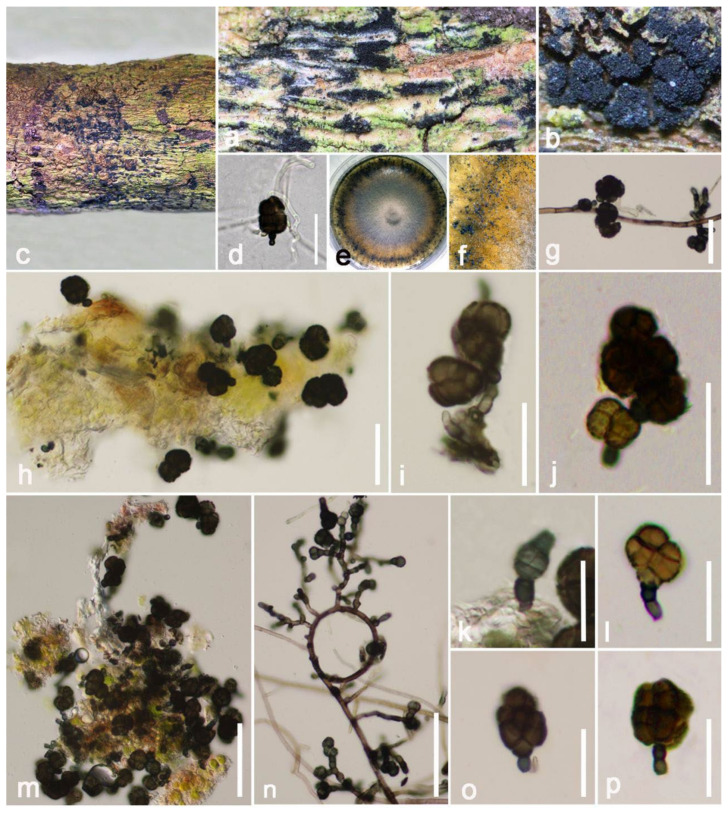
***Paradictyoarthrinium diffractum*** (HKAS 122194). (**a**–**c**) Colonies on host; (**d**) germinating conidia; (**e**) above colony on PDA; (**f**) close-up of colony on PDA; (**g**,**n**) conidium with hypha from PDA; (**i**–**k**) conidia connected conidiogenous cells; (**h**,**m**) close-up conidia raised on host tissue; (**l**–**p**) conidia. Scale bars: (**m**,**n**) = 50 μm; (**d**,**g**,**h**) = 30 μm; (**i**,**j**,**l**–**p**) = 20 μm; (**k**) = 15 μm.

***Paradictyoarthrinium diffractum*** Matsush., Matsushima Mycological Memoirs 9: 18 (1996) (Figure 12).

Index Fungorum number: IF 415849; Faces of Fungi number: FoF 01854.

*Saprobic* on decayed branch of *Mangifera indica*. **Sexual morph**: Undetermined. **Asexual morph**: *Colonies* on natural substrate, black, superficial, scattered, gregarious, powdery. *Mycelium* 2–3 μm wide, immersed, composed of pale brown to dark brown, septate, branched. *Conidiophores* reaching 5–10 μm long, 1–3 μm diameter, erect, macronematous, slightly flexuous, short, branched to unbranched, raised from hyphae, septate, slightly constricted at the septum, brown to green-brown, thick-walled. *Conidiogenous cells* 3–4.5 μm ($\bar{x}$ = 4 μm, *n*= 20) high, 2–4 μm ($\bar{x}$ = 3 μm, *n* = 20) wide, monoblastic, integrated, terminal, determinate, pale brown. *Conidia* 10–25 × 10–15 μm ($\bar{x}$ = 19 × 14 μm, *n* = 30), muriform, solitary or developing in chains, becoming ellipsoidal to irregular in shape, reddish brown to dark brown, turning brown to dark brown on maturity, deeply constricted at the septum, verrucose.

**Culture characteristics**: Conidia germinated on PDA within 18 h; colonies on PDA, circular, effuse, loose, fluffy, velvety, wavy margin, reaching 30 mm after one week at 27 °C, white at center, dark brown to pale brown to margin, brown at reverse side, producing conidia near edge circle. Mycelium 2–3 μm ($\bar{x}$ = 2.5 μm, *n* = 20) wide, tough, superficial, brown, branched, septate. Conidiophore formed from hyphae, septate, straight to slightly curved, mostly unbranched, nearly same width with mycelium. Conidiogenous cells 2–6 × 2–4 μm ($\bar{x}$ = 4 × 3 μm, *n* = 30), holoblastic, terminal. Conidia 10–20 × 10–15 μm ($\bar{x}$ = 17 × 13 μm, *n* = 30), ellipsoidal to irregular, muriform, brown, branched chains, round truncate at base.

**Material examined**: China, Yunnan Province, Baoshan city, Longling county, on decayed branch of *Mangifera indica*, (99°16′80″ E, 25°12′23″ N, 800 m), 27 December 2019, E.F. Yang, MB005 (Herb. HKAS 122194), living culture, KUMCC 21-0336. Genbank numbers: ITS: OL413022, LSU: OL413012, *rpb2*: OL690511.

**Notes**: The comparison of morphological characteristics of colonies and conidiogenous cells show that our isolate is highly consistent with *Paradictyoarthrinium diffractum* in morphologically. However, our strain has slightly olivaceous to brown conidia, while the previous description recorded brown to dark brown conidia [13,63,64]. However, based on LSU, ITS, and *rpb2*, BLASTn results show our isolate has 100% similarity with *P. diffractum* (MFLUCC13-0466, BCC 8704, MFLUCC 12-0557). Our phylogenetic tree topology of the ML and BI analyses were similar to Liu et al. [86], while our isolate clusters together with *P. diffractum* (MFLUCC12-0557, MFLUCC 13-0466) with high bootstrap support (Figure 11). In this study, *Paradictyoarthrinium diffractum* (KUMCC 21-0336) represents a new host record and country record for this fungus (Table 1).

Phaeoseptaceae S. Boonmee, Thambugala and K.D. Hyde, Mycosphere 9(2): 323 (2018).

***Phaeoseptum*** Y. Zhang, J. Fourn. and K.D. Hyde, Mycologia 105: 606 (2013).

Index Fungorum number: IF 561889

**Type species**: *Phaeoseptum aquaticum* Y. Zhang, J. Fourn. & K.D. Hyde, Mycologia 105: 606 (2013).

**Figure 13:**
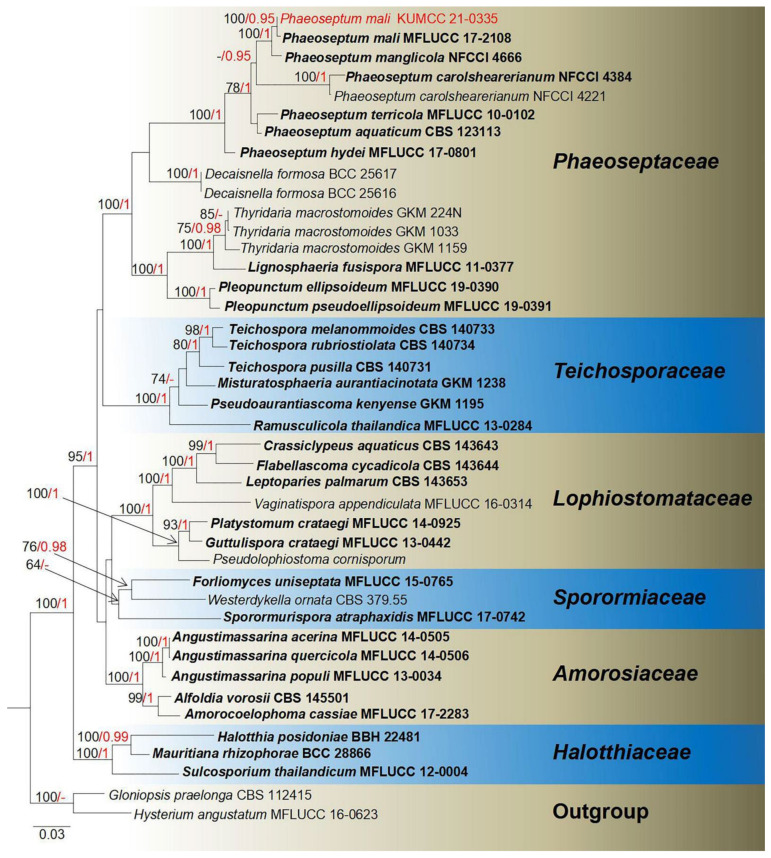
Phylogram generated from maximum likelihood analysis based on a combined LSU, SSU, ITS, and *tef1-α* sequence datasets. Related sequences were taken from Phukhamsakda et al. [65]. The 42 strains are included in the combined gene analyses’ 3348 total characters, including gaps (LSU: 1–848 bp, SSU: 849–1874 bp, ITS: 1875–2427 bp, *tef1-α*: 2428–3348 bp). Tree topology of the ML analysis was similar to the BI. The matrix had distinct alignment patterns, with the final ML optimization likelihood value of −20,978.655356 (ln). All free model parameters were estimated by RAxML model, with 1323 distinct alignment patterns and 24.92% undetermined characters or gaps. Estimated base frequencies were as follows: A = 0.239024, C = 0.256186, G = 0.275083, T = 0.229707, with substitution rates AC = 1.071457, AG = 2.395085, AT = 1.478858, CG = 1.122178, CT = 6.702940, GT = 1.000000. The gamma distribution shape parameter alpha = 0.570195, and the Tree-Length = 1.698997. The final average standard deviation of split frequencies at the end of total MCMC generations was calculated as 0.008933 in BI analysis. The species determined in this study are indicated in red. Bootstrap values greater than 60% (ML, left) and Bayesian posterior probabilities (BI, right) greater than 0.95 are given at the nodes; hyphens (-) represent values less than 60% in ML/0.95 in BI.

**Notes**: *Phaeoseptum* was first introduced by Zhang et al. [78] with the type species *P. aquaticum*, which was found on submerged branch of *Robinia pseudoacacia* in France [78]. This genus was initially placed in family Halotthiaceae but later was transferred to Phaeoseptaceae [88]. There are six records in Index Fungorum [38]. This genus is characterized by immersed, subglobose, papillate, ostiolate ascomata, trabeculate pseudoparaphyses, mostly 8-spored asci (excluding *P. hydei* with 32 ascosporesin asci), cylindrical-clavate asci with a small ocular chamber or apical ring when it is immature, fusiform, light brown to brown ascospores, dictyosporous with thickened transversal septa, range of 1–3 seriate [60,77,87]. In this genus, only the sexual morph has been reported from China, France, India, and Thailand, while habitats are mostly freshwater, estuarine, or various terrestrial plants [65,78,88,89,90]. The phylogeny of *Phaeoseptum* and closely related genera is shown in Figure 13.

**Figure 14:**
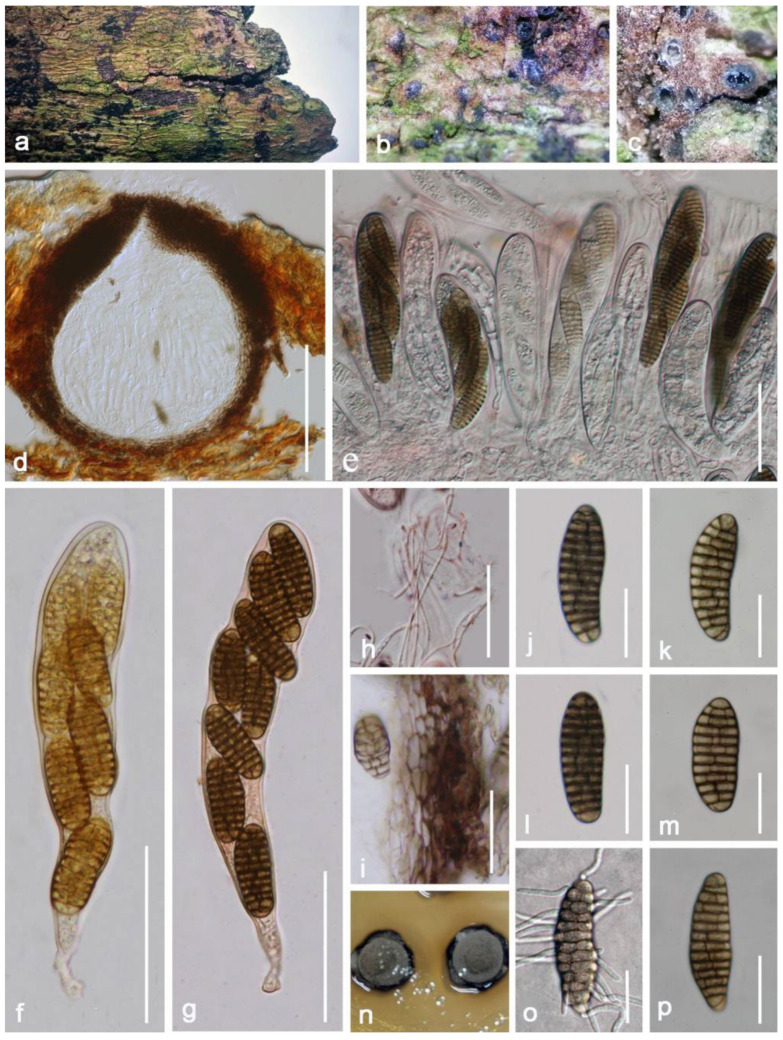
***Phaeoseptum mali*** (HKAS 122193). (**a**) Ascomata on dead brunch of *Mangifera indica*; (**b**,**c**) close-up of substrate; (**d**) section of ascoma; (**e**–**g**) immature and mature asci stained by congo red; (**h**) pseudoparaphyses stained by congo red; (**i**) peridium; (**j**–**m**,**p**) ascospores; (**n**) colonies on PDA; (**o**) germinated ascospore. Scale bars: (**d**) = 150 μm; (**f**,**g**) = 50 μm; (**h**) = 40 μm; (**i**,**o**) = 20 μm; (**j**–**m**,**p**) = 15 μm.

***Phaeoseptum mali*** Phukhams. and K.D. Hyde, Asian Journal of Mycology 2: 120 (2019) (Figure 14).

Index Fungorum number: IF 556265; Faces of fungi number: FoF 05982.

*Saprobic* on decayed branch of *Mangifera indica*. **Sexual morph**: *Ascomata* 260–360 × 210–340 μm ($\bar{x}$ = 308 × 275 μm, *n* = 20), completely immersed under pseudoclypeus, dark-brown to black, scattered to gregarious, globose to oval, conspicuous at the surface, appearing as black prolonged bump on host surface, ostiolate. *Ostiole* canal 50–65 × 60–76 μm, central, short, reddish-brown to brown. *Peridium* 25–55-μm wide, thick-walled, wide at the apical parts, comprising several cell layers of *textura prismatica*; inner layers pale brown cells; middle layers brownish; and outer layers dark brown and cells fuse with host tissue. *Hamathecium* 1–2-μm wide, fusiform, filamentous, septate, branched, embedded in a gelatinous matrix, pseudoparaphyses. *Asci* 140–170 × 20–30 μm ($\bar{x}$ = 157 × 24 μm, *n* = 20), 6–8-spored, bitunicate, short pedicel, club-like, apically rounded, with a minute ocular chamber, thick-walled. *Ascospores* 30–40 × 8–13 μm ($\bar{x}$ = 35 × 11 μm, *n* = 30), overlapping uni or biseriate, ellipsoid, hyaline when young and becomes yellowish to brown at maturity, muriform, with 11–14-transverselly septate, 2 seriate, with a vertical septum in nearly all median cells, not constricted at the septum, smooth-walled, without mucilaginous sheath or appendages. **Asexual morph**: Undetermined.

**Culture characteristics**: Ascospores randomly produced germ tubes from all directions on PDA after 20 h. Colonies slow-growing on PDA, reaching 10-mm diameter after a half month at 27 °C, colonies embedded in medium, circular, umbonate, gray from above with black at margin, clear margin with slightly sunken, highly dense; reverse dark brown, without pigments produced in PDA.

**Material examined**: China, Yunnan Province, Baoshan city, Longling county, on decayed branch of *Mangifera indica* (99°16′80″ E, 25°12′23″ N, 800 m), 27 December 2019, E.F. Yang, MB001 (Herb. HKAS 122193), living culture, KUMCC 21-0335. Genbank numbers; ITS: OL413027, LSU: OL413028, SSU: OL527729, *tef1-α*: OL690512.

**Notes**: Our isolate fits well with *Phaeoseptum mali* that was introduced by Phukhamsakda et al. [65] from *Malus halliana* in Yunnan, China. Our new isolate *P. mali* KUMCC 21-0335 overlaps with *Phaeoseptum mali* (type strain MFLU 19–0406) in asci size (140–170 × 20–30 μm vs. 85–190 × 19–32 μm) and ascospore size (30–40 × 8–13 μm vs. 27–38 × 8–13 μm) [65]. Moreover, the BLASTn expected values and percent-sequence identities of our isolate (ITS, LSU, SSU, and *tef1-α*) result in high similarity (>99%) with *Phaeoseptum mali* (MFLU 19–0406). The phylogenetic trees also showed our new isolate clusters together with *P. mali* with high bootstrap support (Figure 13). Therefore, our isolate identified as *Phaeoseptum mali* with new host record based on morphological features and phylogenetic analyses (Table 1).

Torulaceae Corda, in Sturm, Deutschl. Fl., 3 Abt. (Pilze Deutschl.) 2: 71 (1829).

***Torula*** Pers., Annalen der Botanik (Usteri) 15: 25 (1794).

Index Fungorum number: IF 10248.

**Type species**: *Torula herbarum* (Pers.) Link, Magazin der Gesellschaft Naturforschenden Freunde Berlin 3: 21 (1809).

**Figure 15:**
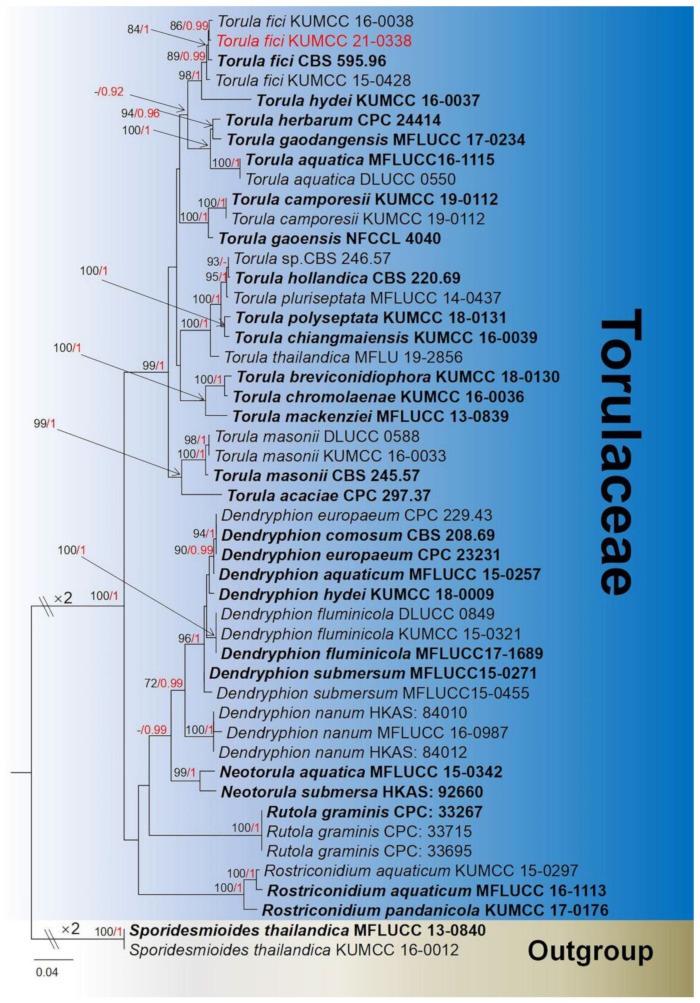
Phylogram generated from maximum likelihood analysis based on a combined LSU, SSU, ITS, *tef1-α,* and *rpb2* sequence datasets. Related sequences were taken from Mapook et al. and Jayasiri et al. [16,59]. The 48 strains are included in the combined gene analyses’ 4399 total characters, including gaps (LSU: 1–882 bp, SSU: 883–1924 bp, ITS: 1925–2493 bp, *tef1-α*: 2494–3345 bp, *rpb2*: 3346–4399 bp). Tree topology of the ML analysis was similar to the BI. The matrix had distinct alignment patterns, with the final ML optimization likelihood value of −18,975.972644 (ln). All free model parameters were estimated by RAxML model, with 1271 distinct alignment patterns and 38.80% undetermined characters or gaps. Estimated base frequencies were as follows: A = 0.245918, C = 0.260831, G = 0.271420, T = 0.221831, with substitution rates AC = 1.945850, AG = 3.692944, AT = 1.530449, CG = 1.028873, CT = 8.723392, GT = 1.000000. The gamma distribution shape parameter alpha = 0.433431, and the Tree-Length = 1.385685. The final average standard deviation of split frequencies at the end of total MCMC generations was calculated as 0.009827 in BI analysis. The species determined in this study are indicated in red. Bootstrap values greater than 60% (ML, left) and Bayesian posterior probabilities (BI, right) greater than 0.95 are given at the nodes; hyphens (-) represent values less than 60% in ML/0.95 in BI.

**Notes**: *Torula* was introduced by Persoon [91] with *T. herbarum* as the type species. The genetic species with similar morphological characteristics are widely distributed all over the world and are mostly known as saprobic on various terrestrial plants and also from submerged decaying wood in rivers or streams [68]. Recently, 541 records were listed in Index Fungorm [38], but only 18 species contain molecular data [66,92]. To date, only sexual morphs have been reported, with micro- or macronematous conidiophores erect from host surface or aerial, with or without apical branch, connected with doliform to ellipsoid, brown, smooth to verruculose, mono- to polyblastic, cupulate conidiogenous cells, branched conidial chains and catenate conidia, appearing globose to subglobose, septate, constricted at the septum, smooth to verrucose, pale brown to dark brown, unequal pigments [59,66,91,92]. The phylogeny of *Torula* and closely related genera is shown in Figure 15.

**Figure 16:**
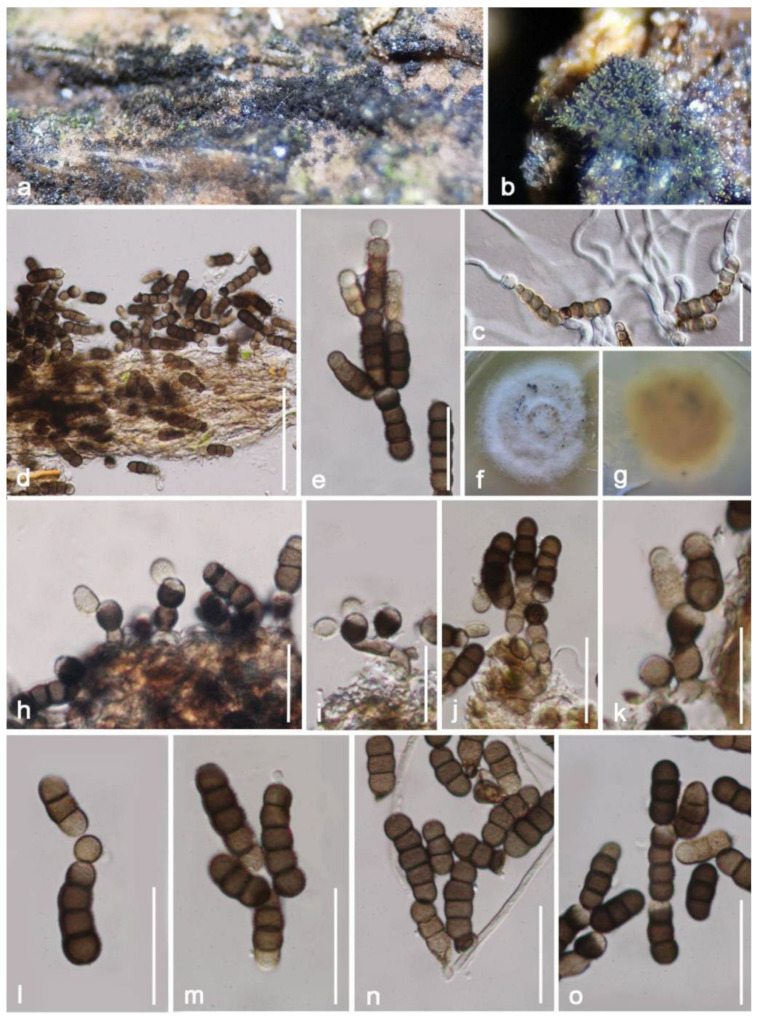
***Torula fici*** (HKAS 122196). (**a**,**b**) Colonies on dead branch; (**c**) germinated conidia; (**d**) colonies on natural substrate under microscope; (**f**,**g**) colonies on PDA; (**h**–**k**) conidiophores with conidiogenous cell; (**e**,**l**–**o**) conidia. Scale bars: (**d**) = 50 μm; (**c**,**e**,**l**–**o**) = 20 μm; (**h**–**k**) = 15 μm.

***Torula fici*** Crous, IMA Fungus 6: 192 (2015) (Figure 16).

Index Fungorum number: IF 816154; Faces of fungi number: FoF 02712.

*Saprobic* on decayed branch of *Mangifera indica*. **Sexual morph**: Undetermined. **Asexual morph**: *Colonies* on the natural substrate effuse, aerial, scattered, dark-brown to black, powdery, easily disconnected when disturbed, hairy. *Mycelium* 1.5–3 μm ($\bar{x}$ = 2 μm, *n* = 20) wide, superficial to immersed, septate, hyaline to brownish, branched hyphae. *Conidiophores* 4–9 × 2–5 μm ($\bar{x}$ = 6.5 × 3 μm, *n* = 10), micronematous, reduced to conidiogenous cells, with hyaline to pale brown, unbranched, subcylindrical to subglobose. *Conidiogenous cells* 3–6 × 3–6 μm ($\bar{x}$ = 4 × 5 μm, *n* = 20), mono to polyblastic, doliform to oval, terminal, dark brown to black, smooth to slightly verruculose, thick-walled. *Conidia* 15–21 × 5–6 μm ($\bar{x}$ = 18 × 6 μm, *n* = 30), phragmosporous, greyish-brown to dark brown, verruculose, septate, constricted at the septa, round at both ends, composed of globose to subglobose, some produced in branched chains, easily separating, unequal pigments, shallower at the terminal end.

**Culture characteristics**: Conidia germinated in PDA over one night, rapidly growing, reaching around 40 mm after one month, circular, flat, edge entire, effuse, gray at the center, whitish at the margin; reverse: reddish-brown, becoming white outwardly, without pigment produced in PDA. The sporulation occurs on upper part of PDA, with gregarious conidia sporulating after one month.

**Material examined**: China, Yunnan Province, Honghe Menglong village, on decayed branch of *Mangifera indica*, (102°50′11″ E, 23′41′01″ N, 500 m), 22 December 2020, E.F. Yang, HHE009 (Herb. HKAS 122196), living culture, KUMCC 21-0338. Genbank numbers; ITS: OL413025, LSU: OL413020, SSU: OL413013, *rpb2*: OL765288, *tef1-α*: OL739467.

**Notes**: Based on morphological features, our isolate largely overlaps with *Torula fici* in morphologies, both having effuse, aerial, black-brown colonies, conidiogenous cells mono- to polyblastic, doliform to oval, terminal, cupulate, and they also have the same greyish-brown to dark brown, verruculose conidial chains. The BLASTn results of ITS and *tef1-α* of the new isolate (KUMCC 21-0338) shows similarities of 99.81% and 99.78% with *Torula ficus* (KUMCC 16-0038). Phylogenetic analyses show our new isolate clusters with *T. ficus* (KUMCC 16-0038) with high bootstrap support (Figure 15). Considering the morpho-molecular data analyses, we conclude that our new collection is a new host record on *Mangifera indica*, as *T. fici* has been reported earlier from China but in difference hosts (Table 1).

**Pleosporales genera*incertae sedis***.

***Crassiparies*** M. Matsum., K. Hiray. and Kaz. Tanaka, Fungal Diversity 78: 63 (2016).

Index Fungorum number: IF 815295; Faces of fungi number: FoF 02024.

**Type species**: *Crassiparies quadrisporus* M. Matsum., K. Hiray. and Kaz. Tanaka, Fungal Diversity 78: 63 (2016).

**Figure 17:**
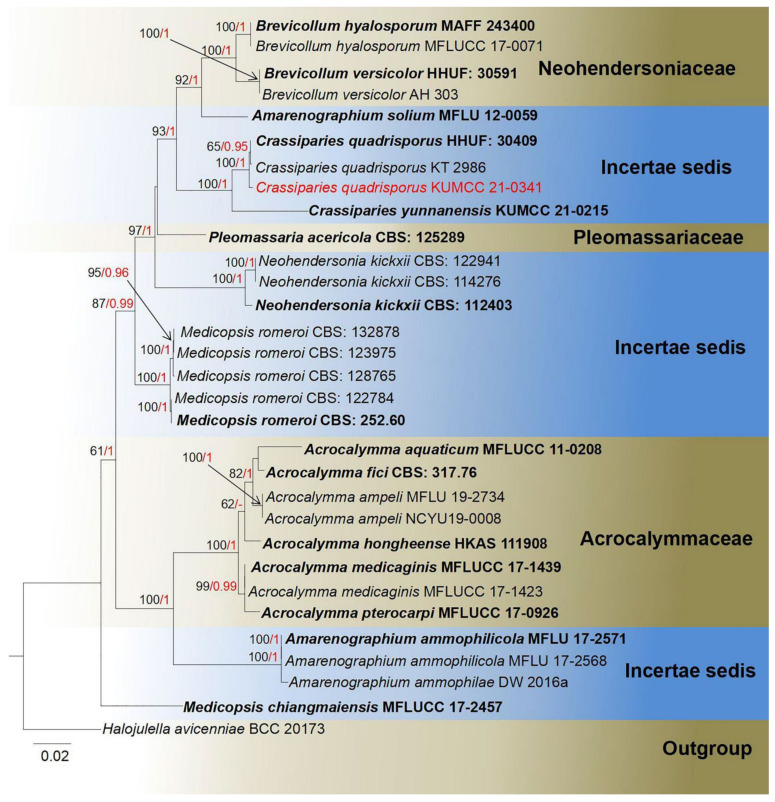
Phylogram generated from maximum likelihood analysis based on a combined LSU, SSU, ITS, and *tef1-α* sequence datasets. Related sequences were taken from Li et al. [69]. The 31 strains are included in the combined gene analyses’ 3240 total characters, including gaps (LSU: 1–888 bp, SSU: 889–1781 bp, ITS: 1782–2329 bp, *tef1-α*: 2330–3240 bp). Tree topology of the ML analysis was similar to the BI. The matrix had distinct alignment patterns, with the final ML optimization likelihood value of −11,009.361241 (ln). All free model parameters were estimated by RAxML model, with 702 distinct alignment patterns and 19.72% undetermined characters or gaps. Estimated base frequencies were as follows: A = 0.242404, C = 0.240804, G = 0.271065, T = 0.245727, with substitution rates AC = 1.229245, AG = 2.590965, AT = 1.688351, CG = 0.503600, CT = 6.760365, GT = 1.000000. The gamma distribution shape parameter alpha = 0.671776, and the Tree-Length = 0.622587. The final average standard deviation of split frequencies at the end of total MCMC generations was calculated as 0.009945 in BI analysis. The species determined in this study are indicated in red. Bootstrap values greater than 60% (ML, left) and Bayesian posterior probabilities (BI, right) greater than 0.95 are given at the nodes; hyphens (-) represent values less than 60% in ML/0.95 in BI.

**Notes**: *Crassiparies* was established by Li et al. [69] with *Crassiparies quadrisporus* as the type species, which was obtained from dead twigs of *Acer* sp. in Japan. *Crassiparies* shares similar morphological characteristics with *Massarina*. Both genera have cylindrical to clavate asci, broadly fusiform 1-septate ascospores; however, *Crassiparies* differs by thick ascomatal walls, without clypei near the ascomatal necks and 4-spored asci [69,93,94]. While revised descriptions of *Crassiparies* were introduced, new records with asexual morphs were reported by Tanaka et al. [70]. The asexual morphs are characterized by discrete, subepidermal, sub-immersed conidiomata, lateral conidiogenous cells, terminal or intercalary, blastic, hyaline, aseptate, straight, ellipsoidal to spherical conidia [70]. Recently, Lu et al. [95] introduced another new species of *Crassiparies*, which was collected on dead wood of coffee in Yunnan, China. The phylogeny of *Crassiparies* closely related genera is shown in Figure 17.

**Figure 18:**
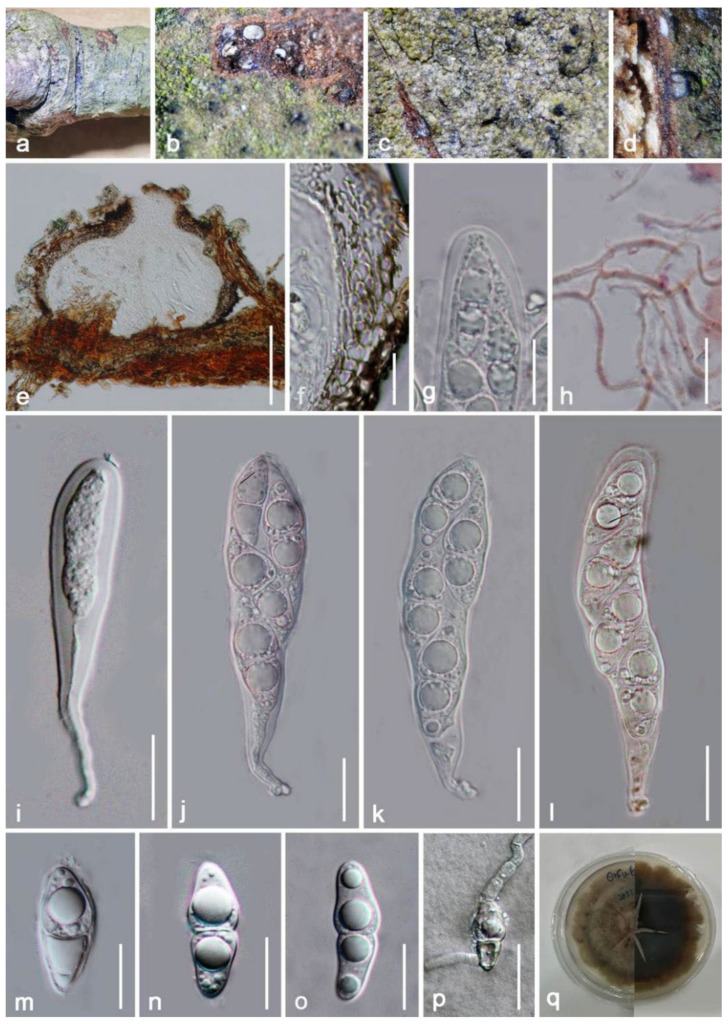
***Crassiparies quadrisporus*** (HKAS 122192). (**a**) Ascomata immersed in the dead wood of *Mangifera indica*; (**b**–**d**) exposed ascoma after section; (**e**) vertical section of ascomata; (**f**) peridium; (**g**) apical chamber of asci; (**h**) pseudoparaphyses strained with congo red; (**i**,**k**) asci; (**j**,**l**) asci stained by congo red; (**m**,**n**) ascospore; (**p**) germinated ascospore; (**q**) colonies on PDA. Scale bars: (**e**) = 150 μm; (**p**) = 30 μm; (**i**–**l**) = 20 μm; (**f**–**h**,**m**–**o**) = 15 μm.

***Crassiparies quadrisporus*** M. Matsum., K. Hiray. and Kaz. Tanaka, Fungal Diversity 78: 63 (2016) (Figure 18).

Index Fungorum number: IF 815295; Faces of fungi number: FoF 02025.

*Saprobic* on decayed branch of *Mangifera indica*. **Sexual morph**: *Ascomata* (include ostile canal) 300–420 μm ($\bar{x}$ = 360 μm, *n* = 20) high, 220–320 μm ($\bar{x}$ = 370 μm, *n* = 20) diameter, ampulliform, solitary to gregarious, brown to black, uniloculate, coriaceous, mostly immersed to completely immersed beneath host epidermis, only visible apical parts are raised up. *Ostiole* 90–120-μm long, 60–80-μm high, dark-brown, cylindrical to subcylindrical. *Peridium* thick-walled, unequal in thickness, exclude 5–15-μm wide at base, other parts fluctuate in the range of 20–30 μm ($\bar{x}$ = 23 μm, *n* = 20), multilayered, comprised of *textura angularis* cells at the inner layers, flat, hyaline, the outer layers are bigger in size and deeper in pigment. *Hamathecium* 1–2-μm wide, hyaline, branched, attached in a gelatinous matrix, filamentous, pseudoparaphyses. *Asci* 90–130 × 15–20 μm ($\bar{x}$ = 110 × 19 μm, *n* = 20), 4-spored, bitunicate, hyaline, apically rounded, broadly cylindrical to clavate, short with club-shaped pedicellate, with clear ocular chamber. *Ascospores* 25–30 × 9–12 μm ($\bar{x}$ = 28 × 10 μm), ellipsoid to fusiform, hyaline, 1–2 septate, obviously constricted at septum, round at both ends, with 2–4 huge oil droplets. **Asexual morph**: Undetermined.

**Culture characteristics**: Ascospores developed on water agar within 24 h, with germ tubes randomly rising. Colonies on PDA are rapidly growing, attaining around 30-mm diameter after two weeks of incubation at 27 °C without light, above: circular, dull, downy, umbonate, brown, radially striated with lobate edge; reverse: with olivaceous brown to black, without pigments produced from PDA.

**Material examined**: China, Yunnan Province, Honghe Menglong village, on decayed branch of *Mangifera indica*, (102°50′11″ E, 23°41′01″ N, 500 m), 22 December 2020, E.F. Yang, erfu6 (Herb. HKAS 122192), living culture, KUMCC 21-0341. Genbank numbers; ITS: OL413011, LSU: OL413015, SSU: OL413026, *tef1-α*: OL739468.

**Notes**: Our new isolate KUMCC 21-0341 shares similar characteristics with *Crassiparies quadrisporus* in (87–110–125 × 17–22 μm vs. 92–131 × 17–21 μm), 4-spored, bitunicate, hyaline, apically rounded, broadly cylindrical to clavate asci, with pedicellate and (27–37 × 9–15 μm vs. 25–30 × 9–12 μm) size, 1–2 septate, broadly fusiform ascospores, with distinct oil droplets when mature [69]. The BLASTn results showed 100% similarity at the ITS (524 bp) and SSU (1024 bp) regions and 99.76% (832/834 bp) similarity at the LSU region with *C. quadrisporus* HHUF: 30409, and the *tef1-α* base pairs comparison yielded up to 99.68% similarity (924/927 bp). Furthermore, the phylogenetic trees also showed our new isolate clusters together with *C. quadrisporus* with high bootstrap support (Figure 17). As the morphological characteristics largely overlap with *C. quadrisporus*, we report our collection as a new host record and country record of *C. quadrisporus* from dead branch of *Mangifera indica* in China (Table 1).

## 4. Discussion

Mango plantations in Yunnan Province, China, represent one of the largest plantation groups in China, contributing to the annual total economic output with CNY 1.8 billion. The area of land under mango cultivation was 74,100 hm^2^ in 2018, and the annual production reaches 0.47 megatons [96]. Baoshan and Honghe are two large mango planting areas in Yunnan, mainly attributable to the suitable temperature and rainfall. Flowering occurs from January to March, and the fruiting period is April to July. The most widely cultivated mango varieties in these areas are Keitt, Guifei, Sannian, Nang Klangwan, and JinHwang [7,97,98,99]. Honghe, located in the dry and hot river valley (north tropical climate regions), has an average annual temperature of 24.9 °C, annual sunshine time of 2300 h, and average annual precipitation of 800–1000 mm, while Baoshan, located in west of Yunnan (south subtropical climate regions), reports an average annual air temperature of 21.5 °C, annual sunshine time of 2300 h, and average annual precipitation of 750–900 mm [99,100,101,102].

Mango, an important and economically useful across the world, is largely cultivated in subtropical to tropical regions, while mango-associated fungi, especially pathogens, are always discovered [103,104,105,106,107]. Although more than 2000 fungal records on mango have been documented, only a few saprobic fungi species associated with mango have been introduced; among the fungi already reported from mango, less fungal species have been reported from the order Pleosporales [12]. In this study, we introduce saprobic fungi in Pleosporales that were collected from Yunnan Province, China. *Mangifericomes* is introduced here as a new fungal genus associated with mango. In addition, further research is needed to confirm whether fungi in mango are “host-specific” or if they can be “host generalists” that jump within different hosts.

## Figures and Tables

**Table 1:** Information of fungi isolated on mango in this study and previous studies.

Order	Family	Species	Host	Distribution	References
**Pleosporales**	Massarinaceae	*Vaginatispora amygdali*	Endocarp of *Amygdalus persica*	Wakayama, Japan	[56]
			Dead branch of *Mangifera indica*	Yunnan, China	This study
	Melanommataceae	*Byssosphaeria siamensis*	Decaying wood of unidentified host	Chiang Rai, Thailand	[61]
			*Pandanus* sp.	Phang Nga, Thailand	[62]
			Dead endocarp of *Mangifera indica*	Yunnan, China	This study
	Neomassariaceae	*Neomassaria hongheensis*	Dead branch of *Mangifera indica*	Yunnan, China	This study
	Parabambusicolaceae	*Paramonodictys hongheensis*	Dead branch of *Mangifera indica*	Yunnan, China	This study
		*Paramonodictys yunnanensis*	Dead branch of *Mangifera indica*	Yunnan, China	This study
	Paradictyoarthriniaceae	*Paradictyoarthrinium diffractum*	Dead twig in stream	Rustenburg, South Africa	[63]
			Spathe of *Cocos nucifera*	Goa, India	[64]
			Dead stumps and stems of *Tectona grandis*	Chiang Rai, Thailand	[13,62]
			Dead branch of *Mangifera indica*	Yunnan, China	This study
	Phaeoseptaceae	*Phaeoseptum mali*	Decaying twigs of *Malus halliana*	Yunnan, China	[65]
			Dead branch of *Mangifera indica*	Yunnan, China	This study
	Torulaceae	*Torula fici*	*Ficus* sp.	Cuba	[66]
			Dead stems of *Chromolaena odorata*	Chiang Rai, Thailand	[16,67]
			Dead leaf of *Pandanus* sp.	Chiang Mai, Thailand	[14]
			Decaying cone of *Magnolia grandifora*	Yunnan, China	[59]
			Decaying fruit pericarp of *Garcinia* sp.	Ranong, Thailand	[59]
			Submerged decaying wood	Yunnnan, China	[68]
			Dead branch of *Mangifera indica*	Yunnan, China	This study
	*Incertae sedis*	*Crassiparies quadrisporus*	Twigs of *Acer* sp.	Mie, Janpan	[69,70]
			Dead branch of *Mangifera indica*	Yunnan, China	This study
		*Mangifericomes hongheensis*	Dead branch of *Mangifera indica*	Yunnan, China	This study

## Data Availability

Not applicable.
